# Thalidomide mitigates Crohn's disease colitis by modulating gut microbiota, metabolites, and regulatory T cell immunity

**DOI:** 10.1016/j.jpha.2024.101121

**Published:** 2024-10-18

**Authors:** Chao-Tao Tang, Yonghui Wu, Qing Tao, Chun-Yan Zeng, You-Xiang Chen

**Affiliations:** a Department of Gastroenterology, Jiangxi Provincial Key Laboratory of Digestive Diseases, Jiangxi Clinical Research Center for Gastroenterology, Digestive Disease Hospital, The First Affiliated Hospital, Jiangxi Medical College, Nanchang University, Nanchang, 330006, China; b Postdoctoral Innovation Practice Base, The First Affiliated Hospital of Nanchang University, Nanchang, 330006, China; c Department of Gastroenterology, Jiangxi Province Hospital of Integrated Chinese and Western Medicine, Nanchang, 330003, China

**Keywords:** Thalidomide, Crohn's disease, FOXP3+ tregs, *Bacteroides fragilis*, 7-Ketolithocholic acid

## Abstract

Thalidomide (THA) is renowned for its potent anti-inflammatory properties. This study aimed to elucidate its underlying mechanisms in the context of Crohn's disease (CD) development. Mouse colitis models were established by dextran sulfate sodium (DSS) treatment. Fecal microbiota and metabolites were analyzed by metagenomic sequencing and mass spectrometry, respectively. Antibiotic-treated mice served as models for microbiota depletion and transplantation. The expression of forkhead box P3^+^ (FOXP3^+^) regulatory T cells (Tregs) was measured by flow cytometry and immunohistochemical assay in colitis model and patient cohort. THA inhibited colitis in DSS-treated mice by altering the gut microbiota profile, with an increased abundance of probiotics *Bacteroides fragilis*, while pathogenic bacteria were depleted. In addition, THA increased beneficial metabolites bile acids and significantly restored gut barrier function. Transcriptomic profiling revealed that THA inhibited interleukin-17 (IL-17), IL-1β and cell cycle signaling. Fecal microbiota transplantation from THA-treated mice to microbiota-depleted mice partly recapitulated the effects of THA. Specifically, increased level of gut commensal *B. fragilis* was observed, correlated with elevated levels of the microbial metabolite 3alpha-hydroxy-7-oxo-5beta-cholanic acid (7-ketolithocholic acid, 7-KA) following THA treatment. This microbial metabolite may stable FOXP3 expression by targeting the receptor FMR1 autosomal homolog 1 (FXR1) to inhibit autophagy. An interaction between FOXP3 and FXR1 was identified, with binding regions localized to the FOXP3 domain (aa238–335) and the FXR1 domain (aa82–222), respectively. Conclusively, THA modulates the gut microbiota and metabolite profiles towards a more beneficial composition, enhances gut barrier function, promotes the differentiation of FOXP3^+^ Tregs and curbs pro-inflammatory pathways.

## Highlights

• THA alleviated DSS-induced colitis in mice by enriching beneficial *Bacteroides fragilis* and reducing pathogenic *Fusobacteriota.*
• *Bacteroides fragilis* increase was attributed to elevated 7-KA levels post-THA treatment.
• 7-KA showed anti-inflammatory action by boosting FOXP3^+^ Treg, possibly stabilizing FOXP3 via FXR1 to curb autophagy.
• FOXP3-FXR1 interaction was identified, with specific binding regions between FOXP3 domain (aa238–335) and the FXR1 domain (aa82–222).

## Graphical abstract

**Figure:**
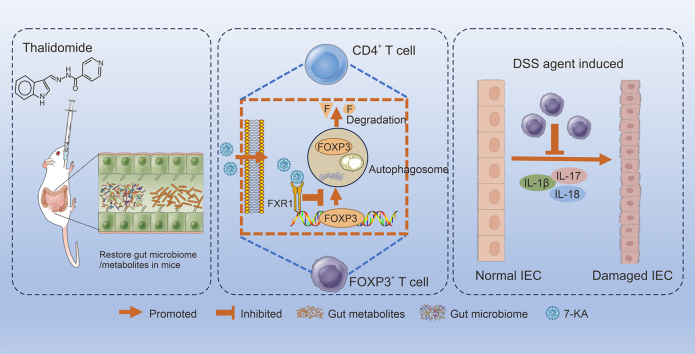

## 1 Introduction

Crohn's disease (CD) is a persistent inflammatory disorder of the gastrointestinal tract marked by a pattern of symptomatic flare-ups and remissions, culminating in intestinal damage and impaired function [1]. The 5-year relapse rate of CD is approximately 55%–85%, which is correlated with hospitalization incidence rates ranging from 0.7 to 4.7 cases per 10,000 individuals [1]. Currently, biologics and immunomodulators constitute the primary therapeutic arsenal for CD; however, their efficacy is limited, underscoring the imperative for the discovery and innovation of more potent therapeutic approaches and intervention strategies.

Thalidomide (THA) gained US Food and Drug Administration (FDA) approval in 1998 as an immunomodulatory drug for the treatment of various neoplastic conditions, including multiple myeloma [2]. Regarding the treatment of CD, an array of clinical trials has demonstrated the efficacy of THA in managing the condition effectively [3]. THA frequently relieves patients with CD with treatment-refractory conditions, particularly those unresponsive to biologic therapies. For example, existing research has highlighted that THA can ameliorate both symptoms and clinical manifestations in such patients, with notable efficacy in those suffering from fistulizing CD [[4], [5], [6]]. Nevertheless, the precise therapeutic mechanisms of action in CD remain elusive and merit further investigation.

Regulatory T cells (Tregs), distinguished by their expression of the transcription factor forkhead box P3 (FOXP3), are extensively recognized for being a highly studied subset of CD4^+^ T cells with immunoregulatory functions capable of fostering tissue homeostasis [7]. Disruption of the T helper cell 17 (Th17)/Treg cell balance is a pivotal factor in the pathogenesis of CD. Th17 cells are primarily involved in instigating and fuelling inflammatory processes, whereas Tregs are instrumental in mitigating established intestinal inflammation. FOXP3, a signature transcription factor for Tregs, governs essential transcriptional programs that are vital for maintaining immune homeostasis [8,9]. FOXP3 is capable of physically interacting with RAR-related orphan receptor gamma t (RORγt), thereby dampening its transcriptional activity. This interaction is crucial for suppressing the generation of interleukin-17 (IL-17) from Th17 cells, effectively curbing the inflammatory cascade [10]. THA notably synergistically enhances CD4^+^ and CD8^+^ T-cell functions through mechanisms such as the activation of protein kinase C within T cells, which in turn increases AP-1 DNA-binding activity. This leads to the induction of IL-2 synthesis and the subsequent activation of downstream signaling cascades, thereby bolstering the T-cell response [11]. The underlying mechanism warrants further exploration for a more comprehensive understanding.

Research has shown that the gut microbiota has the capacity to regulate the emergence of FOXP3^+^ Tregs, which are characterized by their distinctive “inductive” genetic signature. These findings indicate that the microbiota can fortify mucosal immunity by guiding the differentiation of conventional T cells into FOXP3^+^ Tregs, thereby contributing to immune regulation [12]. However, only recently research illuminated how THA specifically modulates the gut microbiota, imparting enduring effects on a spectrum of probiotics and pathogens [13]. Therefore, the network regulatory mechanism governing the interactions of THA, the gut microbiota, and Th17 cell differentiation remains unknown.

In our research, we confirmed the anti-inflammatory role of THA in dextran sulfate sodium (DSS)-induced colitis. Our findings indicate that the therapeutic effect is primarily due to the increased presence of *Bacteroides fragilis* and its bile acid metabolite, 3alpha-hydroxy-7-oxo-5beta-cholanic acid (7-ketolithocholic acid, 7-KA). The metabolite 7-KA facilitates the upregulation of FOXP3^+^ Tregs by preventing the degradation of the FOXP3 transcription factor. Our study reveals a new mechanism underlying the anti-inflammatory action of THA and offers promising therapeutic targets for the treatment of CD.

## 2 Methods

### 2.1 Ulcerative colitis (UC) and CD patients’ samples

In our investigation, biopsy tissues from control subjects and patients with UC and CD were collected via colonoscopy at the First Affiliated Hospital of Nanchang University (Nanchang, China). Informed consent was obtained from all participants, and the study was approved by the Ethics Committee of the First Affiliated Hospital of Nanchang University (Approval number: (2024)CDYFYYLK (08–063) and CDYFY-IACUC-202407QR047). The collected tissues were embedded in paraffin blocks and subsequently subjected to immunohistochemical (IHC) assays to evaluate the expression levels of the proteins FOXP3, IL-1β, and IL-17.

### 2.2 RNA-sequencing analysis

In our study, total RNA was extracted from individual mouse samples using the standard TRIzol protocol provided by Invitrogen (Waltham, MA, USA). The integrity and purity of the RNA were assessed through gel electrophoresis and by employing a NanoDrop spectrophotometer from Thermo Scientific (Waltham, MA, USA). RNA samples, serving as biological replicates, were categorized into two separate pools, ensuring each pool contained an equal number of samples for balanced representation.

Strand-specific RNA-sequencing libraries were constructed using the Illumina TruSeq RNA Sample Preparation Kit, followed by sequencing on the Illumina HiSeq X Ten platform (Illumina, San Diego, CA, USA), conducted by Genergy Biotechnology Co., Ltd. (Shanghai, China). Transcript expression levels were quantified in fragments per kilobase of transcript per million mapped reads (FPKM) using Perl. Differentially expressed transcripts (DETs) were identified through the MA-plot-based method with random sampling (MARS) as implemented in the DEGseq R package, comparing various time points. The DETs were then analyzed for functional and signaling pathway enrichment using Gene Ontology (GO) annotations and the Kyoto Encyclopedia of Genes and Genomes (KEGG) database. Pathways were considered significantly enriched at a *P*-value threshold of less than 0.05, with a minimum requirement of two associated genes for inclusion in the analysis.

### 2.3 Cell flow cytometry

In summary, we initially conducted a longitudinal section of the colonic tissue following a thorough washing to cleanse the surface and the meticulous removal of fat tissues and Peyer's patches. Subsequently, we procured lamina propria lymphocytes from the interphase of a Percoll gradient, employing a washing step with phosphate-buffered saline (PBS) as delineated in our preceding study [14]. Next, the lamina propria cells were stimulated with leukocyte activation cocktail, stained with antibodies, analyzed by flow cytometry. For intracellular staining, cells were fixed and permeabilized with BD Cytofix/Cytoperm (BD, Franklin Lakes, NJ, USA) or transcription factor buffer set according to manufacturer instructions (BD Pharmingen^TM^, Franklin Lakes, NJ, USA). The antibodies, FOXP3 (12-5773-82), CD4 (11-0041-82), and CD25 (17-0251-82), were purchased from Invitrogen (Waltham, MA, USA).

### 2.4 Agents

7-KA was procured from MedChemExpress (HY–W018512; Beijing, China). DSS, with a molecular weight range of 36,000 to 50,000 Da, was acquired from MP Bio Company (Santa Ana, CA, USA) and utilized to induce colitis in our study. To suppress protein synthesis, we employed cycloheximide (CHX; S7418, Selleck Chemicals, Shanghai, China) at a concentration of 20 μm/L, as referenced [15]. Additionally, MG132 (S2619), THA (K17), 3-methyladenine (3-MA; S2767), and chloroquine (S6999) were sourced from Selleck Chemicals. A combination of antibiotics including ampicillin (0.5 g/L), neomycin (0.5 g/L), metronidazole (0.5 g/L), and vancomycin (0.25 g/L) was obtained from Solarbio Science & Technology Co., Ltd. (Beijing, China), and used in our experiments to deplete the gut microbiota.

### 2.5 Cell culture and cell transfection

HEK293T cells (American Type Culture Collection (ATCC), Manassas, VA, USA) were cultured in Roswell Park Memorial Institute 1640 (RPMI-1640) medium (Gibco, Grand Island, NY, USA) supplemented with 10% fetal bovine serum. The cells were maintained in a humidified incubator at 37 °C with an atmosphere containing 5% CO_2_, which is optimal for their growth and viability.

For plasmid transfection, we utilized the Hieff trans liposomal transfection reagent, procured from Yeason Biotechnology Co., Ltd. (Shanghai, China). The transfection process was carried out according to the manufacturer's instructions to ensure high efficiency and minimal cellular toxicity.

We transfected both wild-type and truncated plasmids for the FOXP3 and FMR1 autosomal homolog 1 (FXR1) genes, following a standardized protocol to investigate their roles in cellular processes. All plasmids used in this study were constructed by Obio Technology Co., Ltd. (Shanghai, China), and the complete sequences of these plasmids are detailed in the Supplementary material 1.

### 2.6 Western blot

The detailed process of Western blot was similar as our previous study described [15]. In our study, primary antibodies targeting the following proteins were used: FOXP3 (22228-1-AP, 1:1000; Proteintech, Wuhan, China), FXR1 (13194-1-AP, 1:1000; Proteintech), IL-18 (10663-1-AP, 1:1000; Proteintech), IL-6 (21865-1-AP, 1:1000; Proteintech), IL-17 (66148-1-Ig, 1:2000; Proteintech), IL-1β (16806-1-AP, 1:1000; Proteintech), zonula occludens-1 (ZO-1) (21773-1-AP, 1:1000; Proteintech), glyceraldehyde 3-phosphate dehydrogenase (GAPDH) (1:1000; TransGen Biotech, Beijing, China), Flag-tag (D6W5B, 1:1000; CST, Shanghai, China), HA-tag (C29F4; CST), β-actin (1:1000; TransGen Biotech), sequestosome-1 (SQSTM1/p62) (23214, 1:1000, CST) and microtubule-associated protein 1 light chain 3 (LC3I/II) antibody (14600-1-AP, 1:1000; Proteintech). The secondary antibodies were purchased from TransGen Biotech. We have utilized ImageJ software to quantify the band intensities and have re-evaluated the statistical significance of the observed differences.

### 2.7 Coimmunoprecipitation (Co-IP)

For the Co-IP of endogenous proteins, we employed a direct approach, utilizing primary antibodies to pull down the interacting proteins of interest. In the case of the exogenous Co-IP assay, we engineered wild-type plasmids (HA-FOXP3 and Flag-FXR1) based on the sequences encoding the respective proteins. For the control group, we utilized empty vectors, which served to eliminate the influence of the plasmid backbone on the assay outcomes. The design of the truncated plasmids was informed by the sequences available on the UniProt database, ensuring that our constructs were grounded in accurate and reliable protein sequence data. The concrete process was the same as the previous study [15].

### 2.8 IHC

To eliminate any interfering substances, the tissue sections were treated with anhydrous ethanol for approximately 40 min. This step was followed by antigen retrieval, a process that enhances the exposure of target antigens by incubating the tissue in a citrate solution. Then tissue microarrays were carefully overlaid with primary antibodies and incubated overnight at a controlled temperature of 4 °C. The next day, the microarrays were further processed by incubation with their corresponding secondary antibodies for 1 h at room temperature. Visualization of the targeted proteins was achieved using the 3,3′-diaminobenzidine tetrahydrochloride (DAB) reagent from TransGen Biotech, which provides a distinct color reaction. The nuclei were counterstained with hematoxylin to offer contrast and facilitate the visualization of cellular structures.

The IHC results were evaluated through a meticulous and rigorous assessment conducted by two independent pathologists. They examined the microarrays and assigned scores based on staining intensity and the percentage of positively stained cells. Staining intensity was graded on a scale from 0 to 3, and the proportion of positively stained cells was scored from 0 to 3, with thresholds at 0%, 30%, 60%, and 90%.

Based on these criteria, the expression levels were categorized into three groups: high expression (scores 7–9), moderate expression (scores 4–6), and low expression (scores 0–3). This detailed evaluation process ensured a comprehensive and accurate analysis of the IHC results, yielding valuable insights into the expression patterns of the target proteins within the tissue samples.

### 2.9 Immunofluorescence (IF) assay

HEK293T cells were cultured in chamber slides and allowed to incubate for 24 h. The cells were then fixed using 4% paraformaldehyde for 20 min to preserve their morphology. Permeabilization was followed by using 0.1% Triton X-100 (Solarbio Science & Technology Co., Ltd.) for 1 h. Subsequently, the cells were incubated with a panel of primary antibodies targeting specific proteins: FOXP3 (14-4777-82, diluted 1:100; Thermo Fisher Scientific, Waltham, MA, USA), FOXP3 (22228-1-AP, diluted 1:100; Proteintech), FXR1 (PA5-96481, diluted 1:100 Thermo Fisher Scientific), p62 (18420-1-AP, diluted 1:200; Proteintech), LC3I/II (14600-1-AP, diluted 1:100; Proteintech), CD4 (A26036-PM, 1:200; ABClonal, Wuhan, China), ZO-1 (21773-1-AP, diluted 1:100; Proteintech), IL-1β (16806-1-AP, diluted 1:200; Proteintech), and IL-17 (66148-1-Ig, diluted 1:200; Proteintech). This incubation was performed overnight at 4 °C to ensure optimal antibody binding.

On completion of the primary antibody incubation, the cells were processed with fluorescent secondary antibodies conjugated to goat anti-rabbit or anti-mouse IgG (Thermo Fisher Scientific) to detect the presence of the target proteins. The nuclei were visualized by staining with 4′,6-diamidino-2-phenylindole (DAPI). The IF intensity was assessed using confocal fluorescence microscopy (Olympus, Tokyo, Japan), with excitation at wavelengths of 488 nm and 594 nm.

### 2.10 Reverse transcription-polymerase chain reaction (RT-PCR)

Total RNA from cell was lysed according to the instruction of total RNA Kit (19211ES60, Yeasen Biotechnology, Shanghai, China). Then, the RNA concentration was measured with a NanoDrop 2000 spectrophotometer (Thermo Fisher Scientific). cDNA synthesis was completed according to the instructions of reagents purchased from Yeasen Biotechnology (11141ES10), which included removing residual genomic DNA and preparation of reverse transcription reaction system. Similarly, quantitative PCR was performed with the reagents (Yeasen Biotechnology). The sequence of FOXP3 primer was: 5’-3’, ATGGCACTCAGCTTCTCCTT; 3’-5’, CCAGAGGACTTCCTCAAGCA. GAPDH was used as control. The primer of *B. fragilis* and *Bacteroides* was synthesized by Shenggong Bioengineering Co., Ltd. (Shanghai, China). The 16S was used as control.

### 2.11 Electron microscopy analysis

To assess autophagosome formation, cells were initially seeded in 35 mm culture dishes. Following treatment with 7-KA for 6 h, cells were harvested using a PBS solution. Subsequently, cells were fixed using glutaraldehyde, which was sourced from Solarbio Science & Technology Co., Ltd., to preserve cellular structures for further analysis.

Static bright-field images were captured utilizing a Leica XSP-8CA microscope (Leica, Shanghai, China), providing an initial assessment of cellular morphology and the presence of autophagic structures. For a more detailed examination of autophagosomes induced by 7-KA, cells were subjected to transmission electron microscopy (TEM) (Thermo Fisher Scientific) [16].

### 2.12 Mass spectrometry (MS)

HEK293T cells were carefully isolated and lysed using a non-denaturing lysis buffer that is specifically formulated for Co-IP experiments. The FOXP3-interacting proteins were precipitated using an anti-FOXP3 antibody in combination with protein A/G magnetic beads (Proteintech), following the methodology described in previous research [17]. After the precipitation, the protein fraction was separated and stained according to established protocols, ensuring consistency with procedures used in earlier studies. The stained proteins were then prepared for further analysis. The resulting protein samples were subjected to liquid chromatography-tandem mass spectrometry (LC-MS/MS) using a Thermo Fisher instrument (Thermo Fisher Scientific). Additionally, tandem mass tag (TMT) analysis was performed to quantify the relative protein levels and assess the changes in protein expression. The TMT analysis was conducted and meticulously scrutinized by Luming Biotechnology (Shanghai, China) [18].

### 2.13 Establishment and identify of DSS-induced colitis model in mice

The establishment of the DSS-induced acute colitis model followed the methods outlined in a previously published study [19]. Male C57BL/6 mice, aged 6–8 weeks and procured from Beisai Model Biotechnology Co., Ltd. (Suzhou, China), were housed in accordance with stringent guidelines for animal care and welfare.

**Fig. 1:**
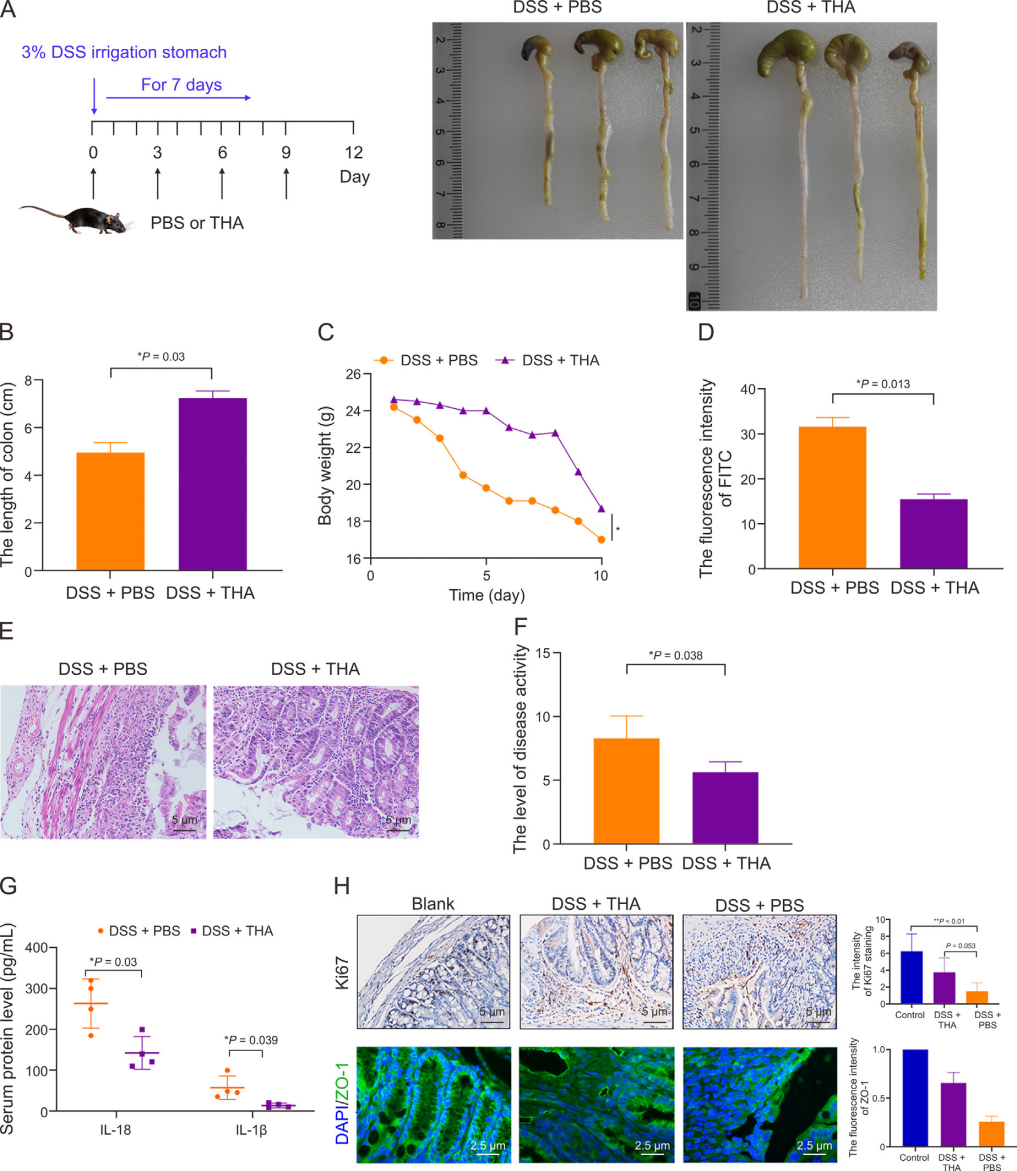
Effect of thalidomide (THA) on dextran sulfate sodium (DSS)-induced colitis mouse model *in vivo*. (A) The methods of medicine irrigation and representative image of colons from mice model. (B) Statistical analysis of colon length across groups. (C) Body weight changes in mice determined once per day from day 0 to day 10 before and after DSS administration and THA administration. (D) Intestinal permeability assessed by fluorescein isothiocyanate (FITC)-dextran assay. (E, F) Macroscopic changes in the colons of mice (E) and the score of disease activity (F) from different gou . (G) The level of interleukin (IL)-18 and IL-1β in the serum of mice measured by enzyme-linked immunosorbent assay (ELISA) in the two groups. (H) The protein levels of Ki67 and zonula occludens-1 (ZO-1) examined by immunohistochemical (IHC) and immunofluorescence (IF) assay, respectively, and the quantitative analysis was shown. Data are presented as the mean ± standard error of the mean (SEM). ^∗^*P* < 0.05, ^∗∗^*P* < 0.01 vs control group.

Each experimental cohort was initiated after a one-week acclimation period. Mice were randomly assigned into two principal groups: (i) the DSS group, and (ii) the DSS + THA group, as depicted in Fig. 1A. Colitis was induced by incorporating 3% DSS into the drinking water for a period of 7 days. Concurrently, from day 0, THA was administered orally to the DSS + THA group at a dosage of 100 mg/kg.

Throughout the study, we closely monitored the mice for changes in body weight, the presence of hematochezia, and fecal score to assess the severity of colitis. On day 10, the animals were humanely euthanized, and serum and colon tissue samples were collected for subsequent analysis.

For the creation of microbiota-depleted mice, a regimen of intestinal cleansing was initiated at 5 weeks of age. Guided by established literature [14], a cocktail of antibiotics, ampicillin (0.5 g/L), neomycin (0.5 g/L), metronidazole (0.5 g/L), and vancomycin (0.25 g/L), was administered for two weeks to achieve a thorough intestinal decontamination.

In the fecal microbiota transplantation experiment, *B. fragilis* (ATCC25285) (Testobio Co., Ltd. Ningbo, China) was cultured *in vitro*, harvested, and resuspended in a solution of 30% glycerol/PBS. After centrifugation, the supernatant was adjusted to an optical density (OD) of 600 nm = 1.0 and stored at −80 °C until required [14].

For the colonization, intestinally cleansed mice and microbiota-depleted mice were randomly divided into two groups. One group received *B. fragilis*, while the control group received an equivalent volume of PBS. Additionally, the DSS + 7-KA group was administered 7-KA via oral gavage at a dosage of 50 mg/kg.

### 2.14 Histological analysis of colitis and disease activity index

As detailed in our previous study [20], colon tissues were fixed in a 4% phosphate-buffered formaldehyde solution for 36 h, followed by embedding in paraffin. Paraffin sections of 5 μm thickness were carefully prepared and stained with hematoxylin and eosin (HE) to visualize cellular structures and inflammation.

The degree of inflammation in the tissue was meticulously evaluated using a light microscope at magnifications of ×40 and ×100 (Olympus). The severity of inflammation was graded according to the well-established system developed by Truelove and Richards [21], which provides a standardized method for assessing histological changes in inflammatory conditions.

To determine the disease activity index, a comprehensive assessment was conducted, incorporating both the percentage of weight loss and the viscosity of stool. As demonstrated by previous research, weight loss and stool consistency are categorized into four grades [22]. In this study, the disease activity score was calculated by aggregating the scores for weight loss percentage with those for stool viscosity, providing a composite measure of disease severity.

### 2.15 Fluorescein isothiocyanate (FITC)–dextran intestinal permeability assay

As described by previous study [23], mice were fasted for 3 h before the assay and then administered by oral gavage with 150 μL FITC-dextran (Sigma-Aldrich, St. Louis, MO, USA) (80 mg/mL). 4 h later, 100 μL of blood was collected and centrifuged at 10,000 *g* for 10 min, and collected serum was diluted 1:4 (*v/v*) in water and 100 μL/well was added in 96-well culture plates. Finally, the FITC-labeled dextran levels were measured by fluorometry with 485 excitation/528 emission.

### 2.16 Enzyme-linked immunosorbent assay (ELISA) assay

We procured the IL-18, IL-17, and IL-1β ELISA kits from Elabscience (Wuhan, China), which were utilized for the *in vitro* quantitative measurement of these cytokines in mouse serum. The procedure was carried out in strict accordance with the manufacturer's instructions. To summarize the process, the mouse serum was initially diluted threefold. A volume of 100 μL of both the standard solutions and the diluted serum samples was dispensed into the designated wells of the ELISA plate. The plate was then sealed and incubated for 90 min at 37 °C. Next, the contents of the wells were carefully decanted, and 100 μL of the working solution was promptly added to each well. A subsequent incubation step of 60 min at 37 °C was conducted. After thorough washing to remove unbound conjugates, 100 μL of the horseradish peroxidase (HRP) conjugated detection antibody was added to each well and incubated for 30 min at 37 °C. Finally, the chromogenic substrate reagent and stop solution were sequentially added to the wells, leading to a colorimetric reaction. The optical density (OD) of each well was measured at a wavelength of 450 nm using a microplate reader.

### 2.17 16S ribosomal RNA (rRNA) amplicon sequencing

Fecal DNA extraction was performed using the MolPure® Stool DNA Kit (18820ES50, Yeasen Biotechnology) in strict accordance with the manufacturer's guidelines. The extraction procedure commenced with sample preprocessing, encompassing cell lysis, impurity removal, and the enzymatic action of proteinase K to digest proteins and facilitate DNA purification.

Following these initial steps, DNA was extracted using a DNA adsorption column, designed to selectively bind and concentrate DNA from the lysed sample. The concentration and integrity of the extracted DNA were evaluated using a NanoDrop 2000 spectrophotometer and confirmed by agarose gel electrophoresis, ensuring the quality and purity of the DNA for downstream applications.

The extracted DNA served as a template for PCR amplification of bacterial 16S rRNA genes with the use of barcoded primers and Takara Ex Taq polymerase (Takara, Shiga Prefecture, Japan). To assess bacterial diversity, we targeted the V3–V4 (or V4–V5) hypervariable regions of the 16S rRNA genes, employing universal primers 343F (5′-TACGGRAGGCAGCAG-3′) and 798R (5′-AGGGTATCTAATCCT-3′) for amplification.

The quality of the resulting amplicons was inspected via agarose gel electrophoresis, and the PCR products were purified using AMPure XP beads (Agencourt, Shanghai, China) to remove any excess primers, nucleotides, or enzymes. Subsequently, a second round of PCR amplification was conducted to enrich the purified amplicons. Raw sequencing data were delivered in FASTQ format and preprocessed using Cutadapt software to identify and remove adapter sequences, ensuring clean and high-quality data for analysis. QIIME2 software was employed for alpha and beta diversity analyses, estimating microbial diversity through robust metrics such as the Chao1 index and Shannon index.

Further analysis to identify significant differences between groups was conducted using the R package, which included a suite of statistical tests such as analysis of variance (ANOVA), Kruskal-Wallis, T test, and Wilcoxon tests. The linear discriminant analysis effect size (LEfSe) method was utilized to compare the taxonomic abundance spectra between groups, providing a powerful tool for discerning differentially abundant features within the microbial communities.

### 2.18 LC-MS/MS analysis

Samples underwent a meticulous pretreatment process, initially involving the application of L-2-chlorophenylalanine, methanol, and acetonitrile. Subsequently, the supernatant from each sample was meticulously collected using crystal syringes, ensuring clarity and purity. This was followed by filtration and transfer to LC vials, ready for analysis via LC-MS.

The metabolomic data analysis was expertly conducted by Luming Biological Technology Co., Ltd. . The raw LC-MS data were processed using Progenesis QI V2.3 software (Nonlinear Dynamics, Newcastle, UK) to perform a series of refinements. Compound identification was meticulously achieved by leveraging precise mass-to-charge ratio (*m/z*) values, characteristic secondary fragments, and isotopic distribution patterns. Databases such as The Human Metabolome Database (HMDB) were utilized to confirm the identity of detected compounds. The dataset was then imported into R software for principal component analysis (PCA) to visualize the distribution of samples and assess the overall stability of the analytical process. Advanced statistical methods, including orthogonal partial least-squares-discriminant analysis (OPLS-DA) and partial least-squares-discriminant analysis (PLS-DA), were employed to discern variations in metabolite profiles between different groups.

### 2.19 Statistical analysis

In previous studies, statistical analyses were primarily conducted using SPSS, GraphPad Prism 8 software, and R software. A chi-squared test was employed to explore the correlation between IL-17 expression and IL-1β expression. A two-tailed Student's *t*-test was utilized to compare the control group with the treatment group, as well as to verify the significance of variations in metabolites between groups. Each assay was repeated at least twice. Statistical significance was considered at a *P*-value of less than 0.05.

## 3 Results

### 3.1 THA mediates colitis by modulating the IL-17 signaling pathway

To explore the anti-inflammatory effects of THA within the intestinal milieu, C57BL/6 mice were utilized to establish a colitis model through the administration of 3% DSS for a period of 7 days. As illustrated in Fig. 1A, the mice were randomly assigned to either the DSS + PBS group or the DSS + THA group. On the first day, the DSS + THA group received an oral dose of THA (100 mg/kg) via gavage, followed by daily oral administration. Compared with those in the DSS + PBS group, the colon length and body weight of the mice treated with THA were significantly greater, as depicted in Figs. 1B and C. Notably, 24 h prior to examination, a FITC gavage was conducted, which revealed diminished fluorescence intensity in the blood of the mice in the DSS + THA group, implying a reduction in intestinal barrier damage relative to that in the DSS + PBS group, as shown in Fig. 1D.

Further histological examination of colonic tissues from the DSS + PBS group revealed more severe structural damage, mucosal erosion, and inflammatory cell infiltration than those from the DSS + THA group, as presented in Fig. 1E. Disease activity was monitored through weight loss and stool viscosity, with THA-treated mice demonstrating a decreased disease activity score, indicative of a mitigated colitis phenotype, as shown in Fig. 1F. The serum levels of inflammatory markers, such as il1β and il18, were significantly lower in the DSS + THA group than in the DSS + PBS group, as shown in Fig. 1G. Additionally, protein analysis of mucosal cell viability and barrier integrity revealed reduced Ki67 expression in the mucosal cells of the DSS + PBS group compared with the blank control group, along with diminished ZO-1 expression in both the DSS + PBS and DSS + THA groups, as depicted in Fig. 1H.

**Fig. 2:**
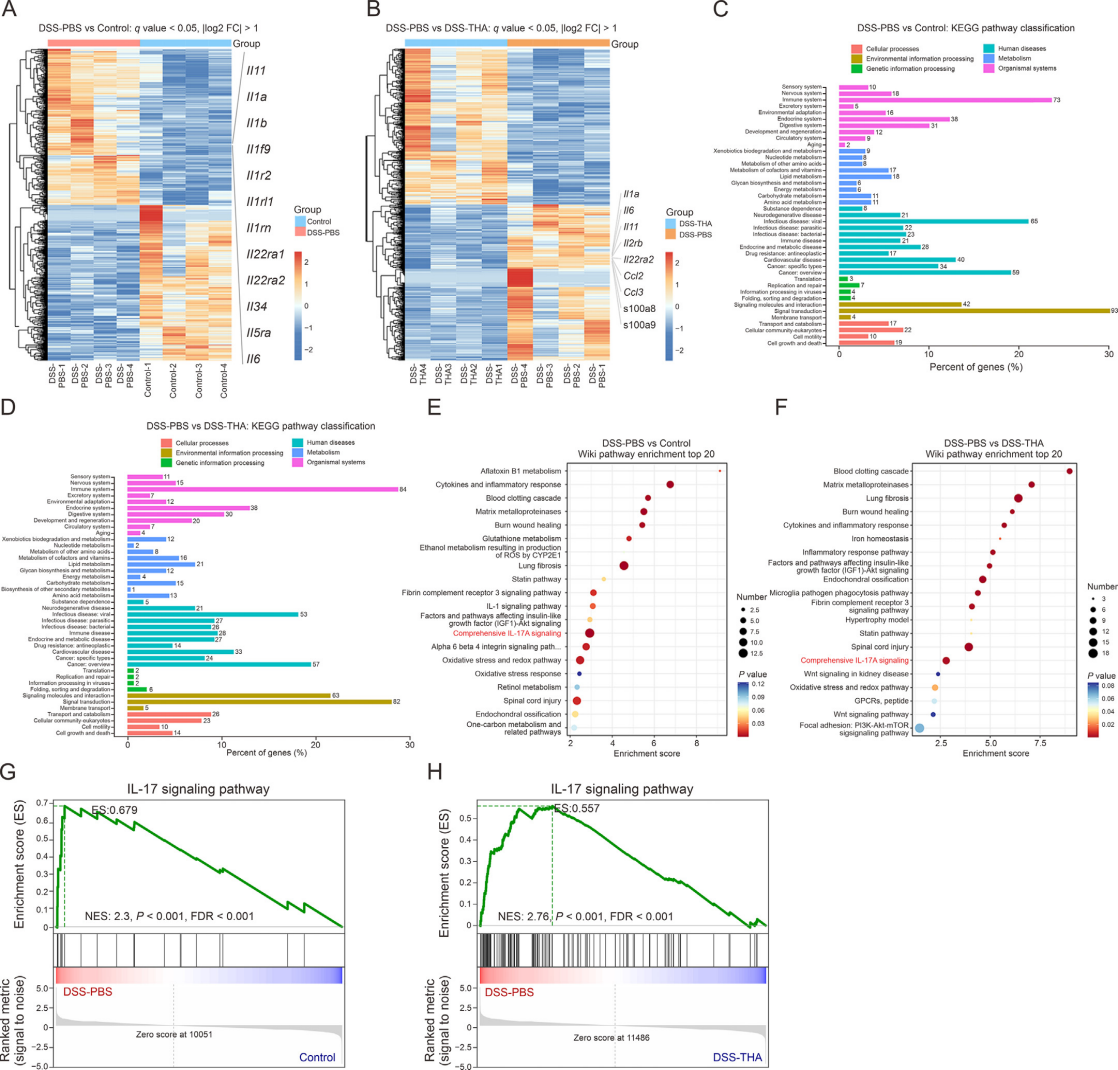
Thalidomide (THA) attenuates the mucosa barrier damage via regulating immune repsonses and interleukin-17 (IL-17) signaling pathway. (A, B) Heatmaps of differentially expressed genes profile between dextran sulfate sodium (DSS)-treated mice and control (A), and DSS-treated and THA-treated mice. (C, D) Kyoto Encyclopedia of Genes and Genomes (KEGG) enrichment analysis of differential genes of DSS-treated mice and control (C) and DSS-treated and THA-treated mice (D). (E, F) Wiki pathway enrichment analysis of differential genes of DSS-treated mice and control (E), and DSS-treated and THA-treated mice (F). (G, H) Gene set enrichment analysis (GSEA) performed on colonic lamina propria cells using the R software tool with normalized enrichment score (NES). DSS-treated mice and control (G), and DSS-treated and THA-treated mice (H). Data are presented as the mean ± standard error of the mean (SEM).

To elucidate the potential mechanisms underlying the impact of THA on colitis progression, RNA sequencing analysis was conducted on mouse tissues, as shown in Fig. 2. In the comparative analysis between the DSS-PBS and control groups, as well as between the DSS-PBS and DSS-THA groups, proinflammatory cytokines, including IL-11, IL-1α, IL-6, and IL-22RA, were identified in the heatmap constructed, indicating their upregulation in DSS-induced colitis tissue, as shown in Figs. 2A and B. As illustrated in Figs. 2C and D, KEGG pathway analysis revealed significant alterations in genes associated with the immune system. Further enrichment analysis revealed that the differentially expressed genes (DEGs) were associated with IL-17 signaling pathways, which was supported by the results of gene set enrichment analysis (GSEA), as demonstrated in Fig. 2E–H.

**Fig. 3:**
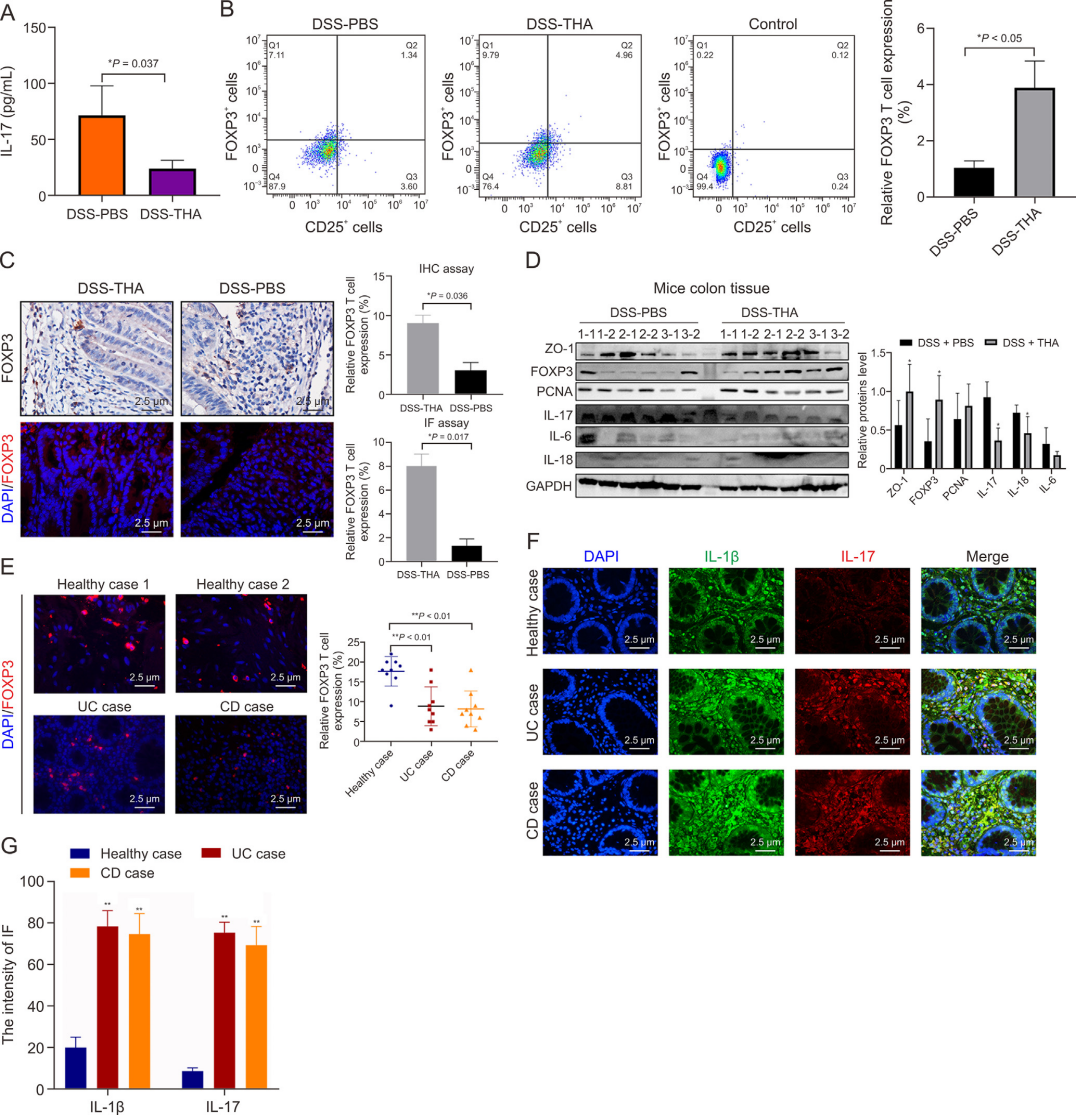
The expression of forkhead box P3^+^ (FOXP3^+^) regulatory T cells (Tregs) in mice colitis model, Crohn's disease (CD), and ulcerative colitis (UC) tissue. (A) The level of interleukin-17 (IL-17) in the serum of dextran sulfate sodium (DSS)-treated and thalidomide (THA)-treated mice measured by enzyme-linked immunosorbent assay (ELISA). (B) Flow cytometry of FOXP3^+^ Tregs (left) and statistical analysis of the percentages of FOXP3^+^ Tregs (right). (C) Immunohistochemical (IHC) and immunofluorescence (IF) assay of FOXP3 protein in colon tissues (left) and quantitative analysis (right). (D) FOXP3, zonula occludens-1 (ZO-1), proliferating cell nuclear antigen (PCNA), IL-18, and IL-6 protein in dextran sulfate sodium (DSS)-treated and thalidomide (THA)-treated mice determined by Western blot assay (left) and the statistical results (right). (E–G) IF staining and quantification of of FOXP3 (E), IL-17 and IL-1β (F, G) in the colon tissue of healthy cases, CD and ulcerative colitis (UC) patients. Data are presented as the mean ± standard error of the mean (SEM). ^∗^*P* < 0.05, ^∗∗^*P* < 0.01. GAPDH: glyceraldehyde-3-phosphate dehydrogenase; DAPI: 4′,6-diamidino-2-phenylindole.

To validate this hypothesis, the serum level of IL-17 was quantified in the mice, and the results revealed lower levels in the DSS + THA group than in the DSS + PBS group (Fig. 3A). Prior research has shown that FOXP3^+^ regulatory Tregs can suppress the production of IL-17 [10]. Flow cytometry analysis of isolated intestinal mononuclear cells revealed a significant decrease in CD4^+^ CD25^+^ FOXP3^+^ T cells in the DSS + PBS group compared with those in the DSS + THA group, as shown in Fig. 3B. IHC and IF experiments on intestinal tissues revealed decreased *Foxp3* expression in the DSS + PBS group, as depicted in Fig. 3C. Western blot analysis revealed increased expression levels of the FOXP3, ZO-1, and proliferating cell nuclear antigen (PCNA) proteins in the intestinal tissues of the DSS-THA group, whereas several proinflammatory factors, such as IL-17, IL-6, and IL-18, were downregulated in the DSS-THA group (Fig. 3D).

Furthermore, bowel tissues from patients with CD and UC were collected for IF assays to measure FOXP3 protein expression. These findings indicated decreased FOXP3 expression in CD and UC tissues, as shown in Fig. 3E. Consistent with this observation, overexpression of IL-1β and IL-17 was noted in UC and CD tissues compared with samples from healthy patients, as illustrated in Figs. 3F and G. These findings suggest that THA may exert anti-inflammatory effects that are independent of the IL-17 signaling pathway.

### 3.2 The gut microbiota is involved in the inhibition of colitis by THA and in the regulation of FOXP3^+^ Tregs differentiation

**Fig. 4:**
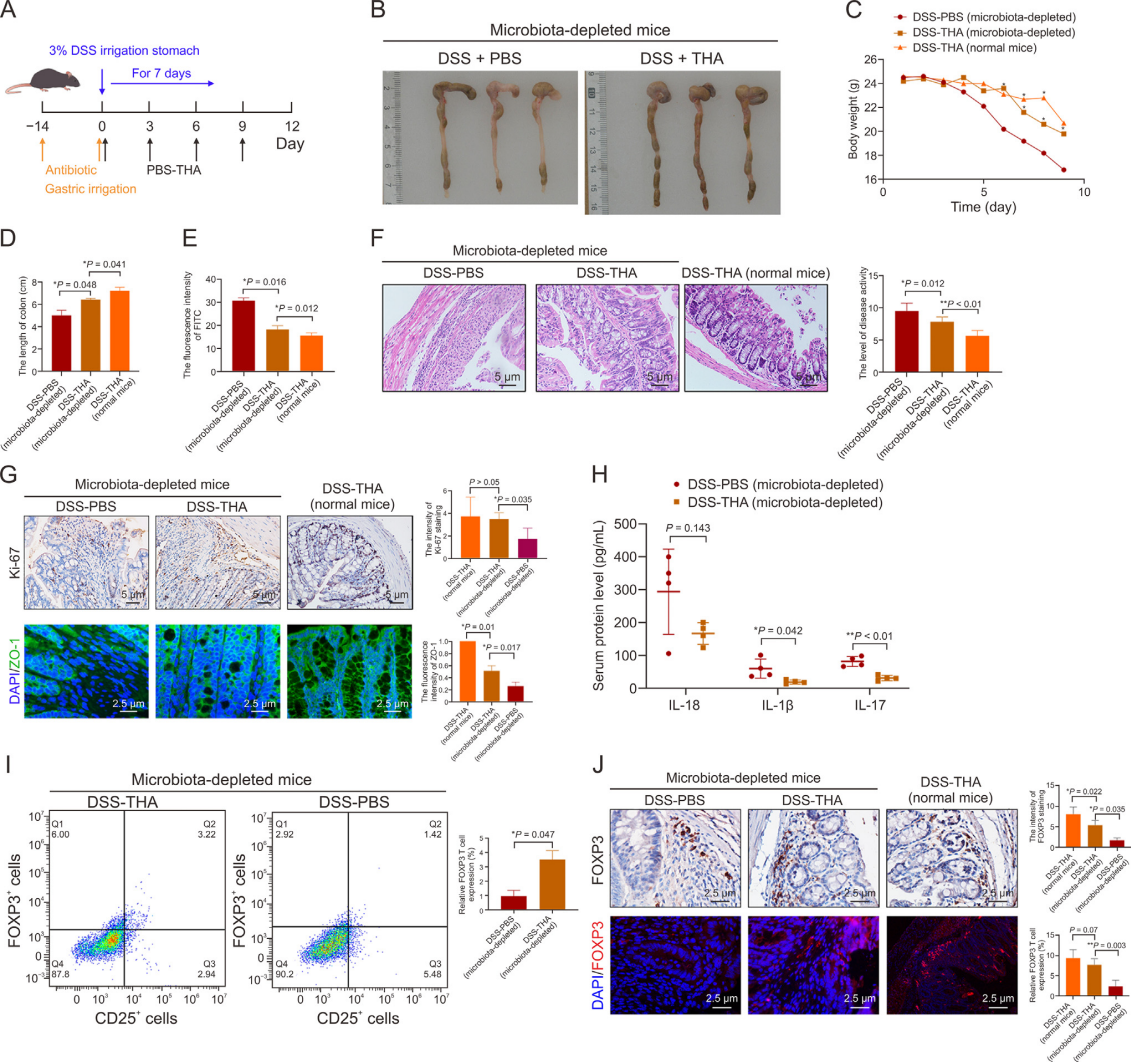
Effects of thalidomide (THA) on dextran sulfate sodium (DSS)-induced colitis microbiota-depleted mice. (A) Schematic description of the animal experimental design. After two weeks of intestinal sterilization treatment, mice were randomly divided into two groups: DSS + PBS group, and DSS + THA group. (B–D) Colon length and body weight change of each group. (E) Intestinal permeability assessed by fluorescence intensity of fluorescein isothiocyanate (FITC). (F) Hematoxylin and eosin (HE) staining of colon sections and disease activity index (DAI) scors. (G) Protein levels of Ki67 and zonula occludens-1 (ZO-1) detected by immunohistochemical (IHC) and immunofluorescence (IF) assay in the microbiota-depleted mice, respectively (left), and quantification analysis (right). (H) interleukin-18 (IL-18), IL-1β, and IL-17 level in the serum measured by enzyme-linked immunosorbent assay (ELISA) assays. (I) Flow cytometry analysis of forkhead box P3 (FOXP3)^+^ regulatory T cells (Tregs) flow cytometry (left) and statistical analysis(right). (J) Representative IHC and IF asaay and quantification of FOXP3 expression in DSS + PBS group and DSS + THA colon tissues from microbiota-depleted mice. Data is presented as the mean ± standard error of the mean (SEM). ^∗^*P* < 0.05, ^∗∗^*P* < 0.01. DAPI: 4′,6-diamidino-2-phenylindole.

To explore the potential interactions among the gut microbiota, THA, and FOXP3^+^ Tregs, C57BL/6 mice were treated with a broad-spectrum antibiotic cocktail. Next, the mice were administered either THA or a PBS control, accompanied by DSS, as outlined in Fig. 4A. Strikingly, microbiota-depleted mice in the DSS + THA group presented notable increases in colon length and body weight compared with those in the DSS + PBS group. However, the anti-inflammatory effect was not more pronounced than that observed in normal mice, as illustrated in Figs. 4B–D. Intriguingly, assessment of FITC fluorescence in the blood revealed that normal mice in the DSS + THA group presented the weakest fluorescence intensity, closely followed by microbiota-depleted mice treated with DSS and THA, as depicted in Fig. 4E.

Consistent with these findings, normal mice treated with DSS and THA presented the lowest colitis scores, superior intestinal cell viability, and the most intact intestinal barrier function, as shown in Figs. 4F and G. Microbiota-depleted mice treated with THA presented intermediate effects, whereas those treated with PBS presented the least favorable outcomes.

Furthermore, lower levels of serum inflammatory cytokines, including IL-1β, IL-17, and IL-18, were detected in microbiota-depleted mice treated with THA, as presented in Fig. 4H. Flow cytometry analyses confirmed a significant decrease in CD4^+^ CD25^+^ FOXP3^+^ T cells among microbiota-depleted mice treated with DSS and PBS, as shown in Fig. 4I. Additionally, IHC and IF assays of intestinal tissues revealed diminished *Foxp3* expression in microbiota-depleted mice treated with DSS and PBS, as shown in Fig. 4J.

**Fig. 5:**
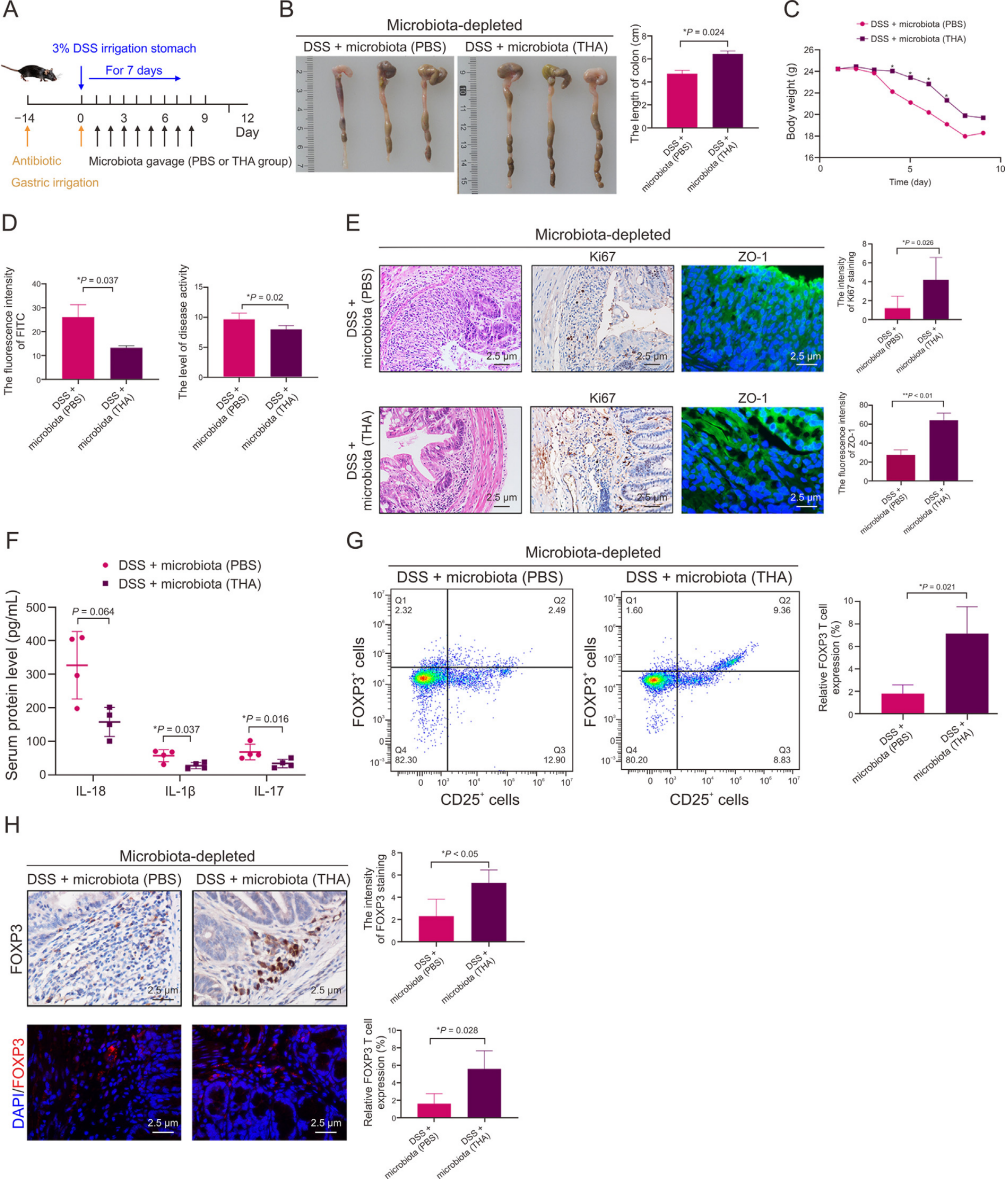
Microbiota from thalidomide (THA)-treated mice mitigated dextran sulfate sodium (DSS)-induced colitis *invivo*. (A) Scheme for the experiment design. Mice were administered antibiotic for 14 days, and then performed microbiota gavage from phosphate-buffered saline (PBS) and THA groups (100 mg/kg). (B) Colon morphology and length of mice receiving microbiota gavage from PBS (vehicle control) or THA groups. (C, D) Body weight changes (C), and fluorescein isothiocyanate (FITC) and disease activity (D) in the mice receiving microbiota gavage from PBS (vehicle control) or THA, respectively. (E) Hematoxylin and eosin (HE) assays of colitis, and immunohistochemical (IHC) and immunofluorescence (IF) assays of zonula occludens-1 (ZO-1) and Ki67 expression and quantification analysis. (F) Interleukin-18 (IL-18), IL-1β and IL-17 level in the serum by enzyme-linked immunosorbent assay (ELISA). (G) Flow cytometry analysis of forkhead box P3 (FOXP3)^+^ regulatory T cells (Tregs) (left) and statistical analysis (right). (H) Immunohistochemical (IHC) and immunofluorescence (IF) assays and quantification of FOXP3 expression in colon tissues . Data are expressed as mean ± standard error of the mean (SEM). ^∗^*P* < 0.05, ^∗∗^*P* < 0.01. DAPI: 4′,6-diamidino-2-phenylindole.

The results of this study highlight that microbiota-depleted mice that received THA exhibited a reduced anti-inflammatory response compared with their normal counterparts treated with THA. To further elucidate the role of the gut microbiota, fecal microbiota transplantation was conducted on donor mice that had been treated with either THA or vehicle control, as depicted in Fig. 5A.

Compared with those receiving microbiota from THA-treated mice, mice transplanted with microbiota from PBS-treated mice experienced pronounced weight loss, increased FITC fluorescence intensity, and increased disease activity scores, as illustrated in Figs. 5B–D. In alignment with these findings, the mice that received microbiota from the PBS-treated group presented increased levels of inflammatory cytokines, including IL-1β, IL-17, and IL-18, along with increased inflammatory cell infiltration and reduced protein expression of Ki67 and ZO-1, indicating a more severe inflammatory state, as shown in Figs. 5E and F.

In line with expectations, the population of CD4^+^ CD25^+^ FOXP3^+^ T cells was significantly enriched in mice that received faecal microbiota from THA-treated mice, as confirmed by IHC and IF assays, as shown in Figs. 5G and H. These outcomes underscore the pivotal function of the microbiota in modulating the anti-inflammatory effects of THA and in regulating the differentiation of FOXP3^+^ Tregs, thereby shedding light on the complex interplay between the gut microbiota and the immunomodulatory actions of THA.

### 3.3 Metabolically active *B. fragilis* suppresses the development of colitis

**Fig. 6:**
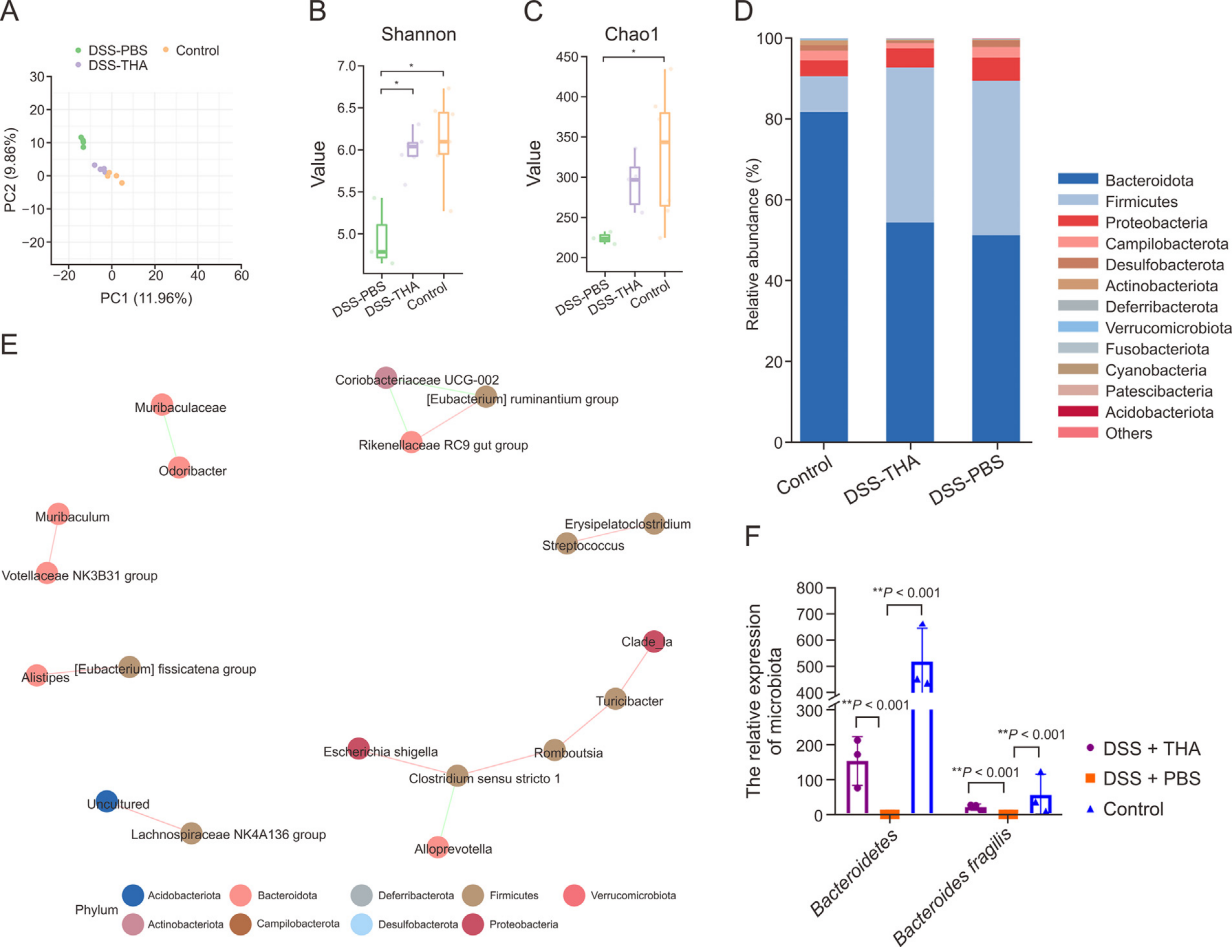
Thalidomide (THA) alters gut microbiota in the stool samples. (A) Principal component analysis (PCA) of stool microbial profiles showing significant differences among control, THA-treated, and dextran sulfate sodium (DSS)-treated mice.. (B, C) α-diversity (Shannon index ) (B) and β-diversity (Chao1 index) (C) of the gut microbiota in three groups mice colitis model. Comparisons of α- and β-diversity were accessed by 2-tailed Mann-Whitney *U* test and permutational multivariate analysis of variance, respectively. (D) Relative abundance of gut microbiome of three groupsanalyzed using a multivariate statistical model. (E) Ecological network among differential bacteria in the three groups. (F) Differential abundance of *Bacteroides fragilis* and *Bacteroidetes* in the colitis model, analyzed by paired Student *t*-test. Data are presented as the mean ± standard error of the mean (SEM). ^∗^*P* < 0.05, ^∗∗^*P* < 0.01 vs control group.

We conducted a comprehensive analysis of the gut microbiota following THA treatment using 16S rRNA sequencing (Fig. 6). PCA revealed significant restructuring of the gut microbiota in response to THA (Fig. 6A). Both α-diversity, which reflects species richness within samples, and β-diversity, which captures species composition differences between samples, were found to increase after THA treatment (Figs. 6B and C). Notably, twelve bacterial taxa exhibited significant shifts due to THA intervention (Fig. 6D). Interestingly, we observed a restoration of *Bacteroidota* in the THA-treated group, which was dysregulated in the non-THA-treated DSS group (Fig. 6D).

Furthermore, intriguing correlations were identified between probiotics and pathogenic bacteria in THA-treated mice, suggesting that THA-enriched probiotics might antagonize pathogenic bacteria in colitis (Fig. 6E). Previous studies have reported significant reductions in the *B. fragilis* population in the stools of patients with inflammatory bowel disease (IBD), with *B. fragilis* playing a crucial role in protecting against inflammatory bowel injury [[24], [25], [26], [27]]. Therefore, we quantified the changes in *B. fragilis* and Bacteroidetes abundances following THA treatment using RT-PCR, which confirmed a stable increase in *B. fragilis* levels (Fig. 6F).

**Fig. 7:**
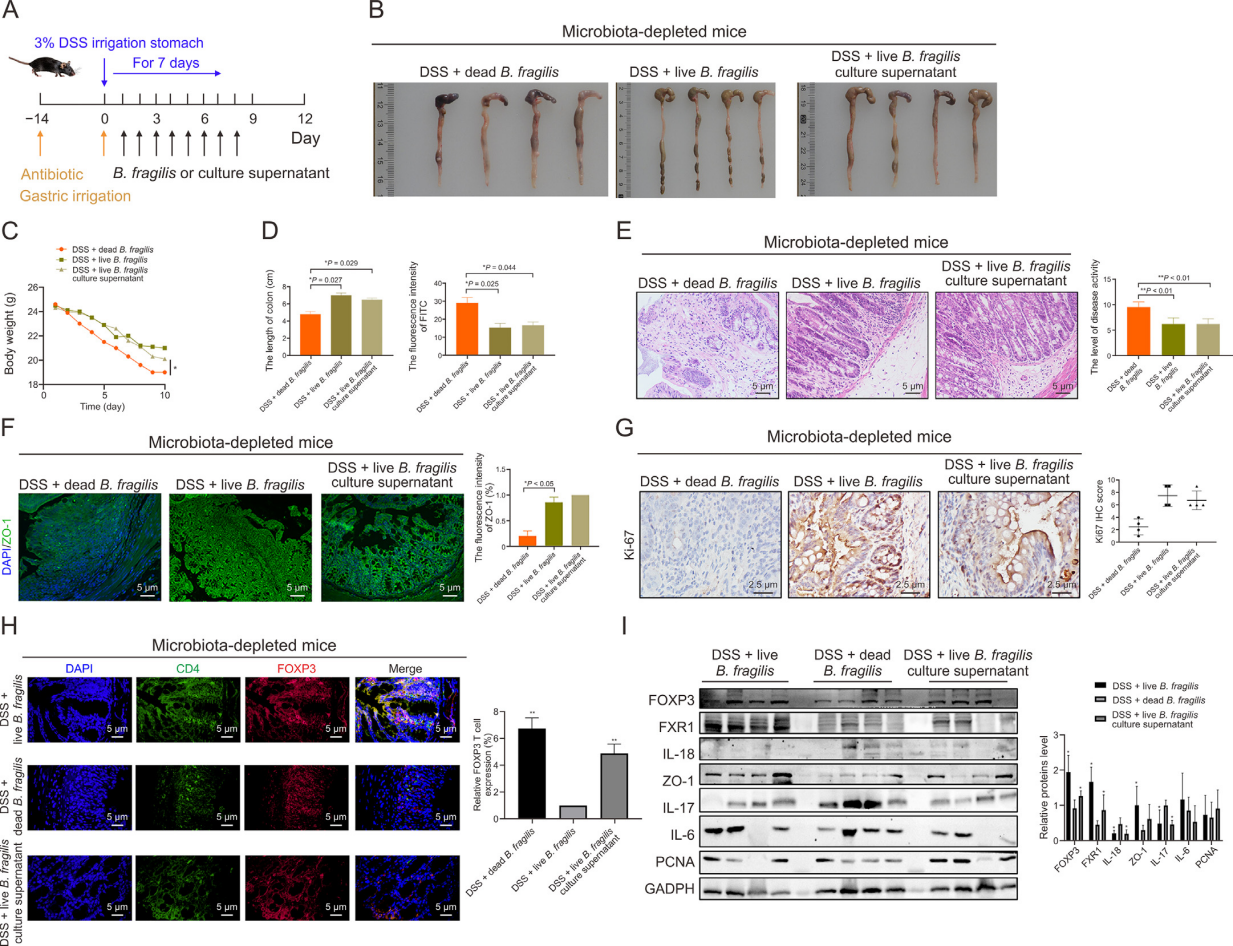
The liver *Bacteroides fragilis* and its supernatant alleviated dextran sulfate sodium (DSS)-induced colitis. (A) Scheme for the experiment design. Mice were administered antibiotic for 14 days, and then performed live *B. fragilis*, dead *B. fragilis* and live *B. fragilis* culture supernatant gastric irrigation per day. (B–D) Colon morphology (B), body weight changes (C), colon length, and fluorescein isothiocyanate (FITC) assay (D) in the mice receiving dead *B. fragilis* enema, live *B. fragilis* and live *B. fragilis* culture supernatant. (E–G) Hematoxylin and eosin (HE) assays (E) of colitis, and immunofluorescence (IF) (F) and immunohistochemical (IHC) (G) assays of ZO-1 and Ki67 expression, respectively and quantification analysis.. (H) IF staining of forkhead box P3 (FOXP3) in colon tissues and quantitative analysis. (I) and statistical results. Data are presented as the mean ± standard error of the mean (SEM), which were analyzed by paired Student *t*-test. ^∗^*P* < 0.05, ^∗∗^*P* < 0.01 vs DSS + dead *B. fragilis*. GAPDH: glyceraldehyde-3-phosphate dehydrogenase; DAPI: 4′,6-diamidino-2-phenylindole.

To evaluate the role of *B. fragilis* in colitis development, we cultured *B. fragilis* *in vivo* and administered it to mice via colonic irrigation following established methods. Additionally, we explored whether its effects were mediated by metabolites rather than the bacterial biomass itself by irrigating the supernatant of *B. fragilis* digested with proteinase K. A schematic of the animal experimental design is depicted in Fig. 7A. As shown in Figs. 7B–D, compared with those in the DSS + live *B. fragilis* or DSS + culture supernatant groups, the mice treated with DSS and dead *B. fragilis* presented shorter and thicker colons, greater severity of weight loss, and increased gut permeability damage. Macroscopic histological examination revealed that, compared with the other two groups, the DSS and dead *B. fragilis* groups presented more severe inflammation, characterized by crypt abscesses, extensive leukocyte infiltration, and increased erosion (Fig. 7E).

Subsequent IHC and IF assays targeting Ki-67 and ZO-1, respectively, revealed decreased levels of these proteins in the mucosal cells of the DSS + dead *B. fragilis* group compared with those in the DSS + live *B. fragilis* and DSS + culture supernatant groups (Figs. 7F and G). These findings collectively indicate that live *B. fragilis* and its culture supernatant ameliorate DSS-induced colitis in mice. IF experiments on intestinal tissues revealed decreased foxp3 expression in the DSS + dead *B. fragilis* mouse group compared with the DSS + live *B. fragilis* and DSS + culture supernatant groups (Fig. 7H). Western blot analysis revealed increased expression levels of the FOXP3, ZO-1, and PCNA proteins in the intestinal tissues of the DSS + live *B. fragilis* group and the DSS + culture supernatant group, whereas several proinflammatory factors, such as IL-17, IL-6, and IL-18, were upregulated in the DSS + dead *B. fragilis* group (Fig. 7I).

### 3.4 7-KA is essential for the anti-inflammatory effects of *B. fragilis*

**Fig. 8:**
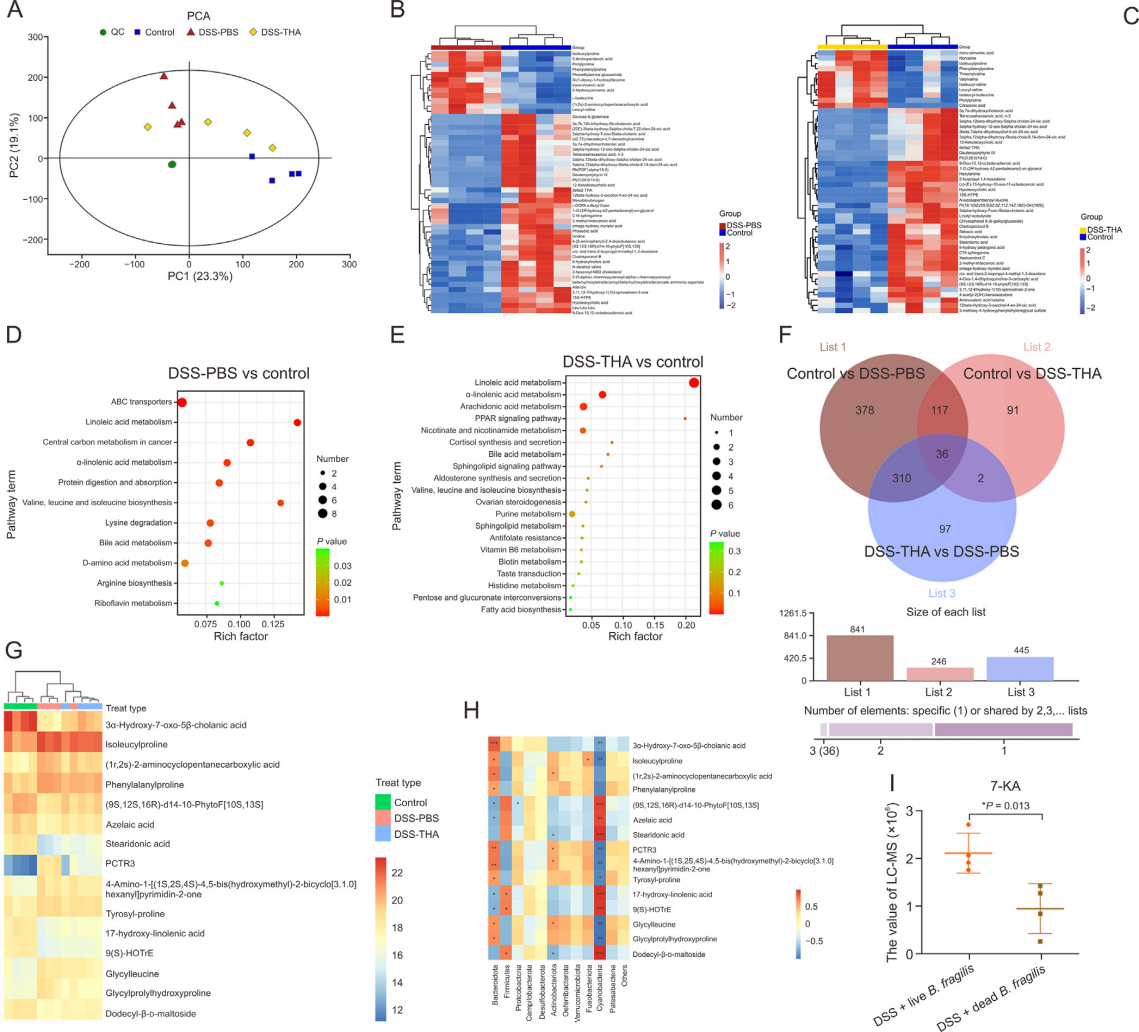
Thalidomide (THA) alters gut microbiota-related metabolites in the stool. (A) Principal component analysis (PCA) of stool metabolic profile showing significantly differences among control, dextran sulfate sodium (DSS), DSS + THA-treated mice colitis model. (B, C) Heatmaps of differential metabolites among control, DSS, and DSS + THA-treated mice (^∗^*P* < 0.05, two-tailed Mann-Whitney *U* test). (D, E) Kyoto Encyclopedia of Genes and Genomes (KEGG) enrichment analysis of differential metabolites in colitis models. (F, G) The Veen plot of co-expressed metabolites (F) and top 15 THA-related metabolites according to the value of fold change (G). (H) Partial Spearman correlation analysis of bacteria and differentially bacteria-related metabolites. (I) Quantification of 3alpha-hydroxy-7-oxo-5beta-cholanic acid (7-ketolithocholic acid, 7-KA) in live and dead *Bacteroides fragilis* transplant groups by liquid chromatography-tandem mass spectrometry (LC-MS)=, data are presented as the mean ± standard error of the mean (SEM), which were analyzed by paired Student *t*-test. ^∗^*P* < 0.05 vs DSS + dead *B. fragilis*.

To identify potential molecular mediators, we conducted LC-MS analysis on faecal samples from mice treated with THA and control mice in the DSS model. PCA distinctly separated the metabolomic profiles of the DSS + THA, DSS + PBS, and control groups (Fig. 8A). The top 50 metabolites with significant differences (*P* < 0.05) were identified between the DSS + THA and DSS + PBS groups relative to the control group (Figs. 8B and C). Notably, our KEGG pathway analysis highlighted enrichment in pathways such as linoleic acid metabolism, bile acid metabolism, and the biosynthesis of valine, leucine, and isoleucine (Figs. 8D and E). A Venn diagram revealed 36 metabolites with significant alterations (*P* < 0.05) across the three groups, and the differences in the top 15 were depicted in a heatmap (Figs. 8F and G). Strikingly, 7-KA levels were significantly lower in the DSS + PBS group than in the DSS + THA group. Additionally, we explored the correlations between the microbiota and metabolites and discovered a positive correlation between 7-KA and *Bacteroidota* (Fig. 8H). MS analysis comparing the DSS + dead *B. fragilis* and DSS + live *B. fragilis* groups revealed significantly higher levels of 7-KA in the DSS + live *B. fragilis* group (Fig. 8I).

**Fig. 9:**
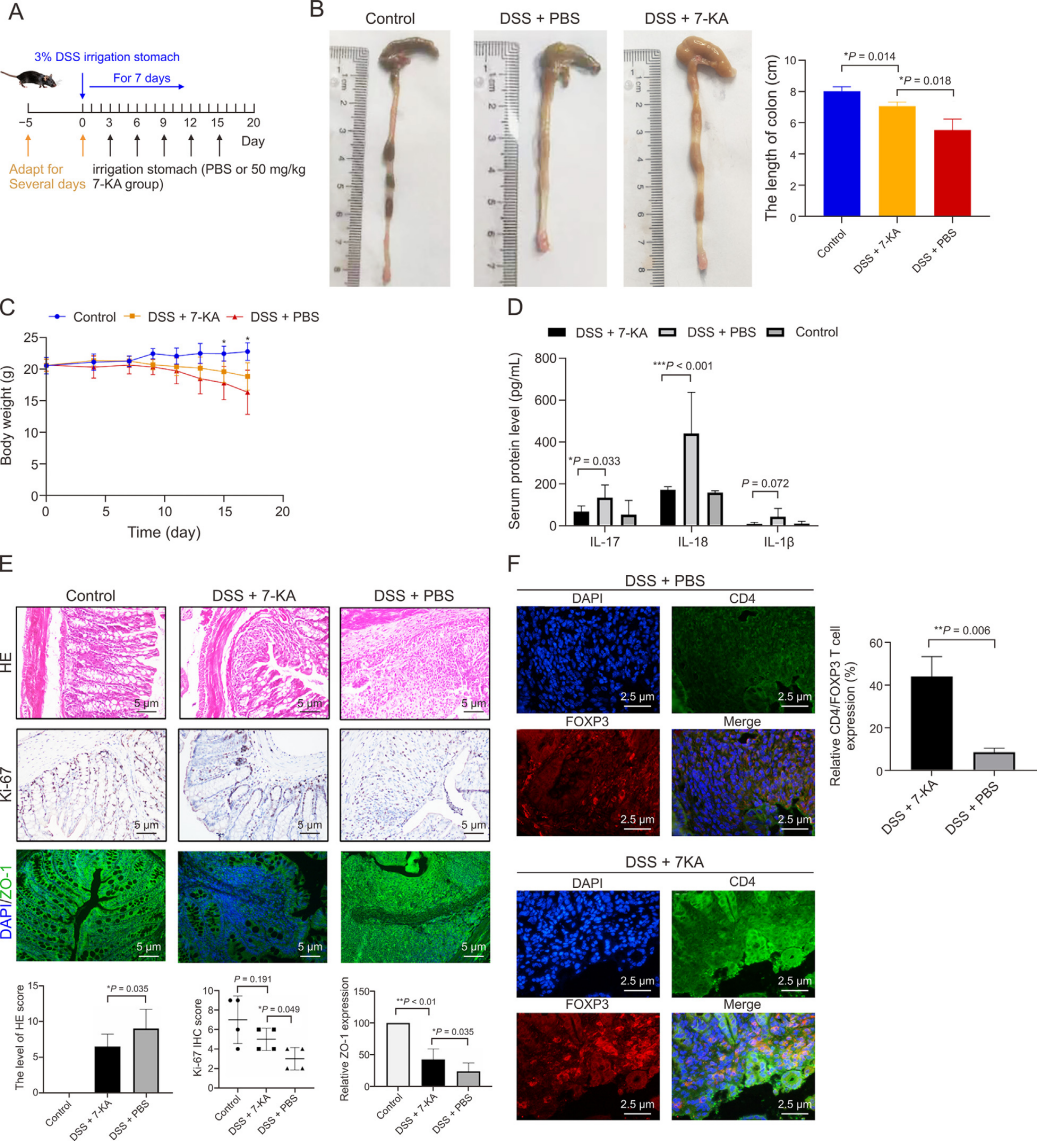
The effects of 3alpha-Hydroxy-7-oxo-5beta-cholanic acid (7-ketolithocholic acid, 7-KA) on mice colitis induced by dextran sulfate sodium (DSS). (A) Induction of colitis, 7-KA-treatment, and assessment timelines are shown. (B, C) Colon length (B) and body weight changes (C) of each group. (D) Serum levels of interleukin-18 (IL-18), IL-1β, and IL-17 measured by enzyme-linked immunosorbent assay (ELISA). (E) Hematoxylin and eosin (H&E) staining for colitis, immunohistochemistry (IHC) for Ki67and immunofluorescence (IF) for zonula occludens-1 (ZO-1) expression and quantificantion. (F) IF co-localization of CD4 and forkhead box P3 (FOXP3) proteins in colon tissues (DSS + PBS vs DSS + 7-KA) and statistical analysis. Data are presented as the mean ± standard error of the mean (SEM), which were analyzed by paired Student *t*-test. ^∗^*P* < 0.05 vs DSS + PBS.

Our data suggest that THA may protect against DSS-induced colitis by promoting the metabolism of 7-KA in *B. fragilis*. To investigate the anti-inflammatory effects of 7-KA, we administered the 7-KA agent (50 mg/kg) to normal control mice and to DSS-induced colitis model mice in the PBS control group (Fig. 9A). Treatment with the 7-KA agent significantly mitigated the reduction in colon length and improved body weight loss (Figs. 9B and C). Moreover, the levels of inflammatory factors such as IL-18, IL-17, and IL-1β were significantly greater in the DSS alone group than in the DSS + 7-KA group (Fig. 9D).

Histological examination of intestinal mucosal tissues revealed that the most severe destruction of mucosal glands occurred in the DSS group, followed by the DSS + 7-KA group, whereas the control group remained intact. The protein levels of ZO-1 and Ki67 were most reduced in the DSS group, followed by those in the DSS+7-KA group (Fig. 9E). Additionally, colocalization fluorescence experiments for CD4 and FOXP3 in intestinal tissues revealed a significant increase in FOXP3 expression in CD4^+^ cells in the DSS + 7-KA group (Fig. 9F). These findings indicate that, compared with DSS-induced colitis, 7-KA treatment leads to an increase in foxp3^+^ Tregs, suggesting that 7-KA protects intestinal barrier integrity and alleviates colitis in mice, potentially through increased *foxp3*^*+*^ Tregs expression.

### 3.5 7-KA promotes the degradation of FOXP3 by targeting the bile acid receptor FXR1

Previous RNA sequencing and *in vitro* studies have established the role of FOXP3^+^ Tregs in the anti-inflammatory effects of THA. Additionally, *B. fragilis* and its metabolite 7-KA have been shown to contribute to the amelioration of gut inflammation by THA. To explore the specific function of 7-KA in FOXP3^+^ Tregs, we conducted *in vivo* assays to assess the influence of 7-KA on FOXP3 expression in 293T cells.

**Fig. 10:**
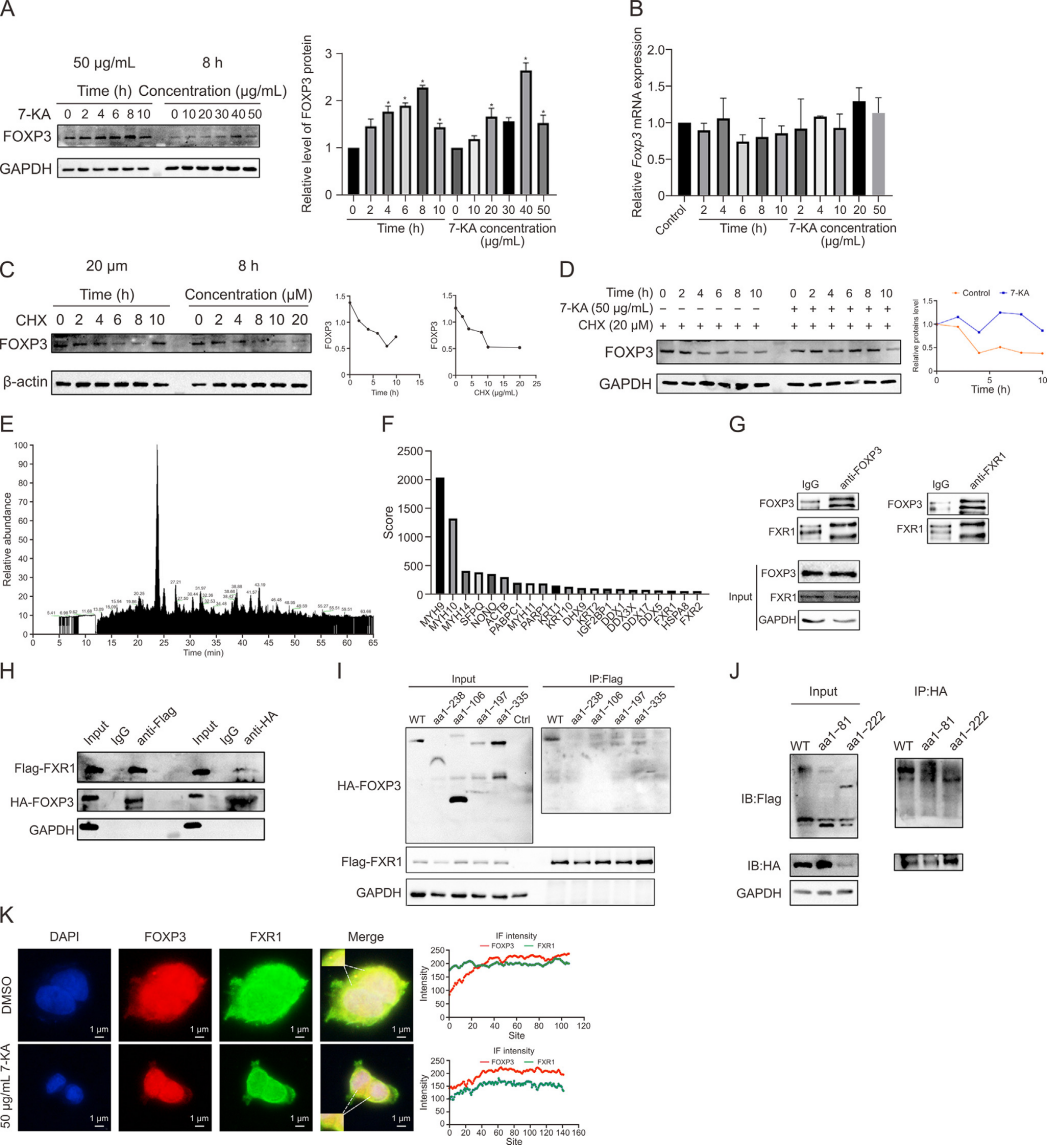
3α,7β,12β-trihydroxy-5β-cholanoic acid (7-ketolithocholic acid, 7-KA) induced the degradation of forkhead box P3 (FOXP3) by functioning on FMR1 autosomal homolog 1(FXR1). (A, B) Western blot (A) and reverse transcription‒polymerase chain reaction (RT-PCR) (B)analysis of FOXP3 expression after 7-KA treatment.Data are presented as the mean ± standard error of the mean (SEM), which were analyzed by paired Student *t*-test. ^∗^*P* < 0.05 vs 0 h. (C) FOXP3 protein degradation in 293T cells treated with cycloheximide (CHX) at varying concentrations or time points. (using the protein at 0 hours as the control) (D) Western blot assay of FOXP3 in 293T cells treated with CHX with 7-KA, and quantification. (using the protein at 0 hours as the control) (E, F) Schematic representation of screening of FOXP3-binding proteins by immunoprecipitation-mass spectrometry (IP-MS). Protein precipitated with IgG served as a negative control. Secondary mass spectrum of peptides showing the identification of FOXP3 protein. (G) Endogenous FOXP3-FXR1 interaction detected by co-immunoprecipitation (Co-IP) in 293T cells using anti-FOXP3 or anti-FXR1 antibodies (IgG as the control). (H) Co-IP of exogenous FOXP3 and FXR1 wild-type (WT) plasmids in transfected 293T cells.(HA-FOXP3: Hemagglutini-FOXP3) (I, J) Truncated FOXP3 and FXR1 plasmid were designed and transfected into 293T cells. Co-IP assay performed to detect the interaction between FOXP3 and FXR1 protein region. (K) Immunofluorescence (IF) analysis of FOXP3-FXR1 colocalization before and after 7-KA treatment. GAPDH: glyceraldehyde-3-phosphate dehydrogenase; DMSO: dimethyl sulfoxide; HA: hemagglutinin.

As depicted in Figs. 10A and B, the treatment of 293T cells with 7-KA led to a time- and concentration-dependent increase in FOXP3 protein levels, whereas *foxp3* mRNA levels remained unchanged. Interestingly, the protein synthesis inhibitor CHX induced FOXP3 protein degradation in a time- and concentration-dependent manner (Fig. 10C), which was significantly attenuated by 7-KA treatment (Fig. 10D).

Given that 7-KA, a bile acid, interacts with specific receptors to exert its effects [28,29], we performed FOXP3 immunoprecipitation followed by MS. This strategy identified FXR1 and FXR2 as FOXP3 interactors (Figs. 10E and F). Western blot analysis confirmed the elevated expression of FXR1 in the intestinal tissues of the DSS + live *B. fragilis* and DSS + culture supernatant groups (Fig. 7I). The interaction between FOXP3 and FXR1 was further validated through a co-IP assay involving both endogenous and overexpressed proteins (Figs. 10G and H). Subsequent Co-IP analyses mapped the interacting domains, highlighting the importance of the FOXP3 domain (aa 238–335) and the FXR1 domain (aa 82–222) for binding (Figs. 10I and J).

**Fig. 11:**
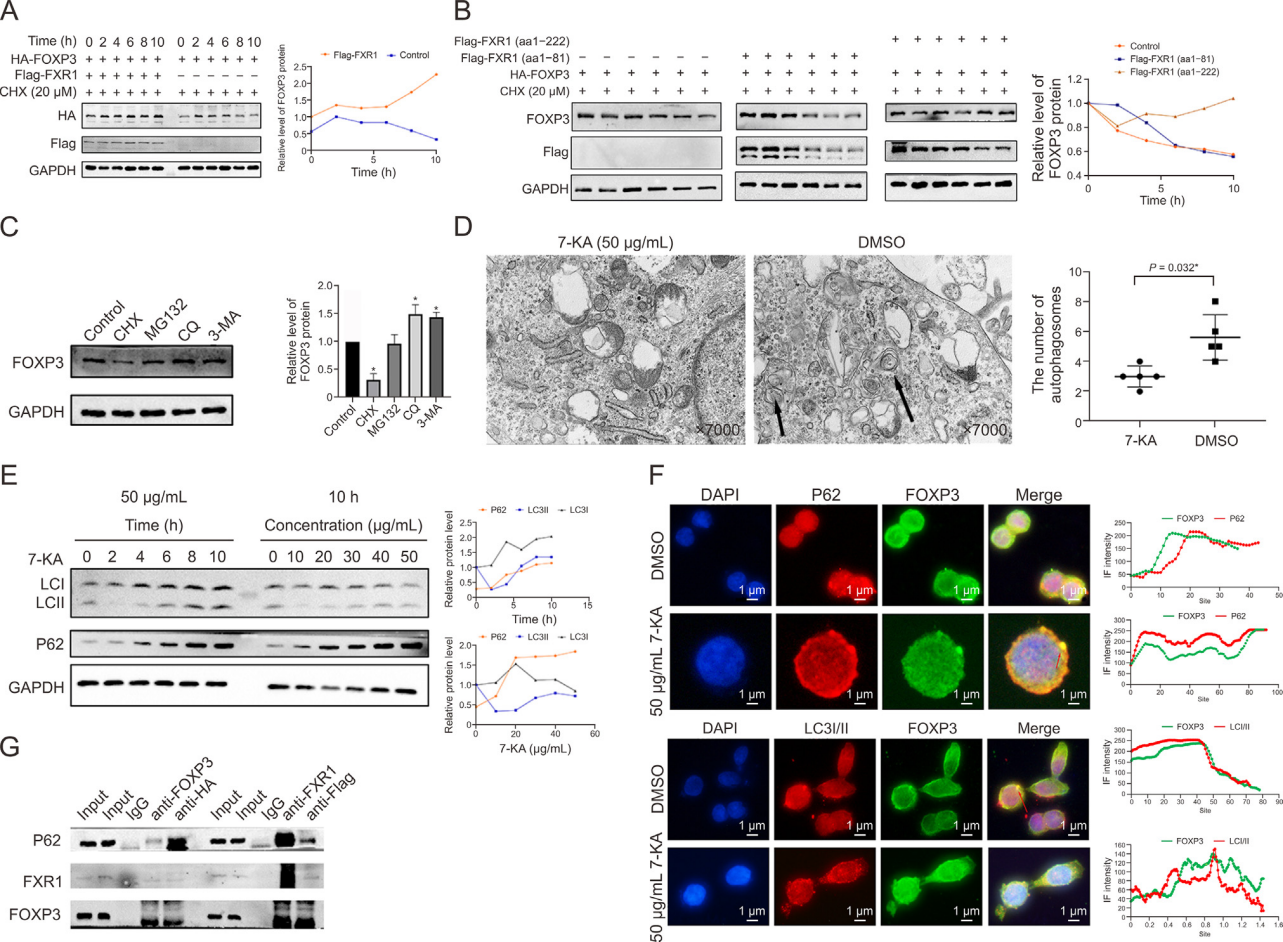
The degradation of forkhead box P3 (FOXP3) protein was associated with FMR1 autosomal homolog 1(FXR1) (aa1–222) and autophagy. (A, B) 293T cells were co-transfected with control vector, FXR1 wide type (WT) plasmid, truncated FXR1 plasmid. Cycloheximide (CHX) chasing assay conducted to testify whether FOXP3 degradation would alter when transfected with FXR1 truncated plasmids, and the statistics analysis. (C) Western blot assay of FOXP3 levels after treating 20 μm CHX, 20 μM MG132, 20 μM chloroquine (CQ) and 20 μM 3-methyladenine (3-MA) for 6 h and the statistics analysis. (D) Autophagy inhibition validated by electron microscopy and the statistical results. (E) Western blot assay of microtubule-associated protein 1 light chain 3 (LC3I/II) and p62 after treated with 3alpha-hydroxy-7-oxo-5beta-cholanic acid(7-ketolithocholic acid, 7-KA). (F) Immunofluorescence (IF) assay of the interaction of FOXP3 with p62 before and after 7-KA treatment, and the statistics analysis. (G) Coimmunoprecipitation (Co-IP) of FOXP3, FXR1 with p62. Data are presented as the mean ± standard error of the mean (SEM), analyzed by paired Student *t*-test. ^∗^*P* < 0.05 vs DMSO or Control.

IF staining revealed the co-location of FOXP3 and FXR1 in the cytoplasm of 293T cells, which was increased by 7-KA treatment (Fig. 10K). FXR1 overexpression was found to inhibit FOXP3 degradation, with the FXR1 domain (aa 1–81) showing no inhibitory effect, whereas the FXR1 domain (aa 1–222) mirrored wild-type FXR1 in function (Figs. 11A and B), suggesting that the FXR1 domain (aa 82–222) may act as an inhibitor of FOXP3 degradation.

Further investigation into the FOXP3 degradation mechanism revealed that the autophagy inhibitor significantly hindered protein degradation, suggesting a role for autophagy in FOXP3 degradation (Fig. 11C). Electron microscopy analysis revealed reduced autophagosome formation following 7-KA treatment (Fig. 11D), with the levels of the autophagy-related marker proteins LC3II and p62 increasing in response to 7-KA treatment (Fig. 11E). IF assays revealed colocalization of FOXP3 with p62 and LC3I/II, with 7-KA treatment enhancing the association with p62 and diminishing that with LC3I/II (Fig. 11F). Co-IP assays confirmed interactions between FXR1, FOXP3, and p62 (Fig. 11G).

These findings collectively suggest that 7-KA protects against DSS-induced colitis in mice by increasing FOXP3^+^ T cell expression, potentially through the inhibition of FOXP3 degradation via autophagy, and by modulating the interaction between FOXP3 and FXR1.

## 4 Discussion

In this study, we have elucidated the anti-inflammatory effects of THA in murine models of colitis, underscoring the pivotal role of a THA-induced microenvironment and immune modulation in disease amelioration.

Prior cohort studies have corroborated the efficacy and tolerability of THA in the management of CD, highlighting its utility in treating early-onset or refractory forms of the condition [30,31]. Regarding its mechanism of action, a recent study identified the involvement of pro-inflammatory cytokines, pro-angiogenic factors, and monocytic cell differentiation in the therapeutic effects of THA, as revealed by large-scale RNA sequencing analysis [31]. Consistent with these findings, our comprehensive analysis of RNA sequencing and ELISA results from mouse serum demonstrated that THA significantly reduced the secretion of pro-inflammatory cytokines, including IL-1β, IL-18, and IL-17. These results suggest that THA exerts an inhibitory effect on inflammation, offering a potent therapeutic strategy for the amelioration of colitis. Based on KEGG and GSEA analyses, we observed that THA modulated key biological processes such as cell growth, apoptosis, and the inflammatory and immune response pathways. These findings are congruent with those reported in other studies, further validating the multifaceted role of THA in regulating immune and inflammatory responses [31]. Pro-inflammatory cytokines, including IL-18, IL-1β, and IL-6, can disrupt the integrity of the mucosal barrier, leading to enhanced intestinal permeability. They also suppress the proliferation of epithelial cells and exacerbate intestinal inflammation, thereby contributing to the progression of inflammatory bowel conditions [[31], [32], [33]].

Our results of bioinformatics analysis, in concordance with existing literature, indicate that the pharmacological mechanisms of THA are closely linked to the IL-17 signaling pathway, predominantly secreted by Th17 cells [34]. Numerous pharmaceuticals and compounds have been documented to modulate the differentiation of Tregs by targeting FOXP3 expression, thereby inhibiting Th17 cell differentiation and IL-17 production [8,35]. In our study, *in vitro* assays revealed that colon tissue treated with THA had an increased proportion of FOXP3^+^ Tregs, which inversely correlated with inflammation. Mechanistically, the gut microbiota plays a pivotal role in mediating THA's effects on promoting FOXP3^+^ Tregs. A recent study has shown that THA modulates the gut microbiota environment in a specific manner, leading to sustained effects on various probiotics and pathogens [13]. Our results demonstrated that DSS treatment reduced the α-diversity and β-diversity of the gut microbiota in mice, whereas the microbiota of THA-treated mice exhibited a significant reversal, with beneficial bacteria such as *B. fragilis* being upregulated post-THA treatment. Further experiments involving *B. fragilis* transplantation confirmed that *B. fragilis*'s efficacy contributed to the alleviation of intestinal inflammation, accompanied by an upward trend in FOXP3^+^ Tregs within the intestinal lamina propria. Indeed, *B. fragilis* secretes the immunomodulatory molecule polysaccharide A (PSA) via outer membrane vesicles, which facilitates the conversion of naïve CD4^+^ T cells into FOXP3^+^ Tregs, ultimately protecting mice from experimental colitis [36].

In our study, we identified that *B. fragilis* could enhance the production of 7-KA, a metabolite that promotes the differentiation of FOXP3^+^ Tregs, representing a significant and innovative concept. However, the absence of a FOXP3 knockout mouse model in our study is acknowledged as a limitation. Future work, with additional funding and support, will focus on constructing a FOXP3 knockout mouse model to further explore the anti-inflammatory mechanisms of *B. fragilis* and 7-KA.

*B. fragilis* is known to negatively regulate the NOD-like receptor family, pyrin domain containing 3 (NLRP3)-mediated inflammatory signaling pathway, inhibiting macrophage activation and the secretion of pro-inflammatory mediators such as IL-18 and IL-1β, thereby reducing intestinal inflammation [37,38]. MS analysis of mouse intestinal content metabolites revealed a significant upregulation of the bile acid metabolite 7-KA in groups treated with THA, with its expression positively correlating with *B. fragilis* levels. Furthermore, *B. fragilis* transplantation increased 7-KA levels, indicating its involvement in alleviating colitis in mice. *In vitro* assays also showed that 7-KA treatment ameliorated colitis, suggesting its role in maintaining intestinal barrier homeostasis and mucosal immunity, such as alleviating aspirin-induced intestinal damage [39,40]. Previous studies suggest that *B. fragilis* can metabolize bile acids through unique bile salt hydrolase (BSH) enzymes, like the BSH gene BF9343_1433 [41,42]. These findings imply that *B. fragilis* may alleviate colonic inflammation through the metabolite 7-KA. However, our experiment lacks a *B. fragilis* BSH knockout strain, preventing confirmation of how *B. fragilis* produces 7-KA.

The action of bile acid metabolites primarily involves their receptors, such as FXR1. For instance, FXR1 activation can regulate the macrophage-T helper cell 1 and T helper cell 17 (Th1/17) axis, reduce the release of inflammatory factors, and possess anti-inflammatory and cell-protective effects [43,44]. In our study, 7-KA was found to inhibit the degradation of FOXP3 by repressing the autophagy process, associated with the bile acid receptor FXR1. Further investigation identified the interacting regions between FOXP3 and FXR1 as the FOXP3 domain (aa 238–335) and the FXR1 domain (aa 82–222), respectively. We propose that 7-KA mediates Tregs differentiation by activating the FXR1 receptor to inhibit autophagy. Inhibition of autophagy is also linked to higher FOXP3 expression and increased differentiation of FOXP3^+^ Tregs [45,46].

## 5 Conclusion

In summary, our study demonstrates, for the first time, the anti-inflammatory effect of THA in colitis *in vivo* by modulating gut microbial composition and promoting the secretion of the protective metabolite 7-KA, which improves gut barrier function and suppresses pro-inflammatory pathways. Beyond its effect on gut microbiota, THA also enhances the differentiation of FOXP3^+^ Tregs *in vitro* by altering the secretion of 7-KA. Our work sheds light on the complex interactions among THA, the gut microbiota, and FOXP3^+^ Tregs, informing future strategies for using THA to treat CD.

## CRediT authorship contribution statement

**Chao-Tao Tang:** Writing – original draft, Resources, Methodology, Investigation, Data curation. **Yonghui Wu:** Writing – original draft, Supervision, Methodology, Conceptualization. **Qing Tao:** Resources, Methodology, Investigation. **Chun-Yan Zeng:** Visualization, Methodology, Investigation, Formal analysis. **You-Xiang Chen:** Writing – review & editing, Supervision, Funding acquisition, Formal analysis.

## Declaration of competing interest

The authors declare that there are no conflicts of interest.
