# Supplementary material for: Thalidomide mitigates Crohn's disease colitis by modulating gut microbiota, metabolites, and regulatory T cell immunity
Source: J Pharm Anal. 2024 Oct 18;15(4):101121. doi: 10.1016/j.jpha.2024.101121 (PMC12041782; doi:10.1016/j.jpha.2024.101121)
Supplement: Multimedia component 1 [file mmc1.docx]

Table 1: The sequence of Primers

| Gene or bacteria | 5’-3’ | 3’-5’ |
| --- | --- | --- |
| 16S | CGGTGAATACGTTCCCGG | TACGGCTACCTTGTTACGACTT |
| Bacteroides fragilis | CACTGAATGCCTGCTCTGTC | TAACAGCTTATCCGGCTGCT |
| Bacteroides | CRAACAGGATTAGATACCCT | GGTAAGGTTCCTCGCGTAT |

2. FOXP3 wild type sequence and truncated plasmid

>NM_014009.4|FOXP3[Human]|CDS 1296bp

atgcccaaccccaggcctggcaagccctcggccccttccttggcccttggcccatccccaggagcctcgcccagctggagggctgcacccaaagcctcagacctgctgggggcccggggcccagggggaaccttccagggccgagatcttcgaggcggggcccatgcctcctcttcttccttgaaccccatgccaccatcgcagctgcagctgcccacactgcccctagtcatggtggcaccctccggggcacggctgggccccttgccccacttacaggcactcctccaggacaggccacatttcatgcaccagctctcaacggtggatgcccacgcccggacccctgtgctgcaggtgcaccccctggagagcccagccatgatcagcctcacaccacccaccaccgccactggggtcttctccctcaaggcccggcctggcctcccacctgggatcaacgtggccagcctggaatgggtgtccagggagccggcactgctctgcaccttcccaaatcccagtgcacccaggaaggacagcaccctttcggctgtgccccagagctcctacccactgctggcaaatggtgtctgcaagtggcccggatgtgagaaggtcttcgaagagccagaggacttcctcaagcactgccaggcggaccatcttctggatgagaagggcagggcacaatgtctcctccagagagagatggtacagtctctggagcagcagctggtgctggagaaggagaagctgagtgccatgcaggcccacctggctgggaaaatggcactgaccaaggcttcatctgtggcatcatccgacaagggctcctgctgcatcgtagctgctggcagccaaggccctgtcgtcccagcctggtctggcccccgggaggcccctgacagcctgtttgctgtccggaggcacctgtggggtagccatggaaacagcacattcccagagttcctccacaacatggactacttcaagttccacaacatgcgaccccctttcacctacgccacgctcatccgctgggccatcctggaggctccagagaagcagcggacactcaatgagatctaccactggttcacacgcatgtttgccttcttcagaaaccatcctgccacctggaagaacgccatccgccacaacctgagtctgcacaagtgctttgtgcgggtggagagcgagaagggggctgtgtggaccgtggatgagctggagttccgcaagaaacggagccagaggcccagcaggtgttccaaccctacacctggcccctga

>NM_014009.4|FOXP3[Human]|Protein 431aa

MPNPRPGKPSAPSLALGPSPGASPSWRAAPKASDLLGARGPGGTFQGRDLRGGAHASSSSLNPMPPSQLQLPTLPLVMVAPSGARLGPLPHLQALLQDRPHFMHQLSTVDAHARTPVLQVHPLESPAMISLTPPTTATGVFSLKARPGLPPGINVASLEWVSREPALLCTFPNPSAPRKDSTLSAVPQSSYPLLANGVCKWPGCEKVFEEPEDFLKHCQADHLLDEKGRAQCLLQREMVQSLEQQLVLEKEKLSAMQAHLAGKMALTKASSVASSDKGSCCIVAAGSQGPVVPAWSGPREAPDSLFAVRRHLWGSHGNSTFPEFLHNMDYFKFHNMRPPFTYATLIRWAILEAPEKQRTLNEIYHWFTRMFAFFRNHPATWKNAIRHNLSLHKCFVRVESEKGAVWTVDELEFRKKRSQRPSRCSNPTPGP

3. FXR wild type sequence and truncated plasmid

>NM_005087.4|FXR1[Human]|CDS 1866bp

atggcggagctgacggtggaggttcgcggctctaacggggctttctacaagggatttatcaaagatgttcatgaagactcccttacagttgtttttgaaaataattggcaaccagaacgccaggttccatttaatgaagttagattaccaccaccacctgatataaaaaaagaaattagtgaaggagatgaagtagaggtatattcaagagcaaatgaccaagagccatgtgggtggtggttggctaaagttcggatgatgaaaggagaattttatgtcattgaatatgctgcttgtgacgctacttacaatgaaatagtcacatttgaacgacttcggcctgtcaatcaaaataaaactgtcaaaaaaaataccttctttaaatgcacagtggatgttcctgaggatttgagagaggcgtgtgctaatgaaaatgcacataaagattttaagaaagcagtaggagcatgcagaattttttaccatccagaaacaacacagctaatgatactgtctgccagtgaagcaactgtgaagagagtaaacatcttaagtgacatgcatttgcgaagtattcgtacgaagttgatgcttatgtccagaaatgaagaggccactaagcatttagaatgcacaaaacaacttgcagcagcttttcatgaggaatttgttgtgagagaagatttaatgggcctggcaataggaacacatggtagtaacatccagcaagctaggaaggttcctggagttaccgccattgagctagatgaagatactggaacattcagaatctacggagagagtgctgatgctgtaaaaaaggctagaggtttcttggaatttgtggaggattttattcaggttcctaggaatctcgttggaaaagtaattggaaaaaatggcaaagttattcaagaaatagtggacaaatctggtgtggttcgagtgagaattgaaggggacaatgaaaataaattacccagagaagacggtatggttccatttgtatttgttggcactaaagaaagcattggaaatgtgcaggttcttctagagtatcatattgcctatctaaaggaagtagaacagctaagaatggaacgcctacagattgatgaacagctgcgacagattggttctaggtcttatagcggaagaggcagaggtcgtcggggacctaattacacctccggttatggtacaaattctgagctgtctaacccctctgaaacggaatctgagcgtaaagacgagctgagtgattggtcattggcaggagaagatgatcgagacagccgacatcagcgtgacagcaggagacgcccaggaggaagaggcagaagtgtttcagggggtcgaggtcgtggtggaccacgtggtggcaaatcctccatcagttctgtgctcaaagatccagacagcaatccatacagcttacttgataatacagaatcagatcagactgcagacactgatgccagcgaatctcatcacagtactaaccgtcgtaggcggtctcgtagacgaaggactgatgaagatgctgttctgatggatggaatgactgaatctgatacagcttcagttaatgaaaatgggctagtcacagttgcagattatatttctagagctgagtctcagagcagacaaagaaacctcccaagggaaactttggctaaaaacaagaaagaaatggcaaaagatgtgattgaagagcatggtccttcagaaaaggcaataaacggcccaactagtgcttctggcgatgacatttctaagctacagcgtactccaggagaagaaaagattaataccttaaaagaagaaaacactcaagaagcagcagtcctgaatggtgtttcataa

>NM_005087.4|FXR1[Human]|Protein 621aa

MAELTVEVRGSNGAFYKGFIKDVHEDSLTVVFENNWQPERQVPFNEVRLPPPPDIKKEISEGDEVEVYSRANDQEPCGWWLAKVRMMKGEFYVIEYAACDATYNEIVTFERLRPVNQNKTVKKNTFFKCTVDVPEDLREACANENAHKDFKKAVGACRIFYHPETTQLMILSASEATVKRVNILSDMHLRSIRTKLMLMSRNEEATKHLECTKQLAAAFHEEFVVREDLMGLAIGTHGSNIQQARKVPGVTAIELDEDTGTFRIYGESADAVKKARGFLEFVEDFIQVPRNLVGKVIGKNGKVIQEIVDKSGVVRVRIEGDNENKLPREDGMVPFVFVGTKESIGNVQVLLEYHIAYLKEVEQLRMERLQIDEQLRQIGSRSYSGRGRGRRGPNYTSGYGTNSELSNPSETESERKDELSDWSLAGEDDRDSRHQRDSRRRPGGRGRSVSGGRGRGGPRGGKSSISSVLKDPDSNPYSLLDNTESDQTADTDASESHHSTNRRRRSRRRRTDEDAVLMDGMTESDTASVNENGLVTVADYISRAESQSRQRNLPRETLAKNKKEMAKDVIEEHGPSEKAINGPTSASGDDISKLQRTPGEEKINTLKEENTQEAAVLNGVS
